# Eater-oriented knowledge framework for reducing salt and dietary sodium intake (scoping review)

**DOI:** 10.3389/fnut.2023.1110446

**Published:** 2023-02-28

**Authors:** Alexandra Endaltseva, Paul Coeurquetin, Thierry Thomas-Danguin, Jean-Pierre Poulain, Laurence Tibère, Anne Dupuy

**Affiliations:** ^1^CERTOP UMR CNRS, University of Toulouse Jean Jaurès, Toulouse, France; ^2^LISST-Cers UMR, University of Toulouse Jean Jaurès, Toulouse, France; ^3^Centre des Sciences du Goût et de l'Alimentation (CSGA), CNRS, INRAE, Institut Agro, Université de Bourgogne Franche-Comté, Dijon, France; ^4^ISTHIA, University of Toulouse Jean Jaurès, Toulouse, France

**Keywords:** public health, dietary sodium, database, salt, interdisciplinary approach to nutrition

## Abstract

Salt and dietary sodium are ubiquitously present in daily food practices and, at the same time, reducing salt intake presents an important public health issue. Given such an ambivalent position of salt in human diet, we argue that public health guidelines toward dietary sodium reduction require an eater-oriented knowledge framework. In this article we are making the first steps toward a flexible interdisciplinary database which would include nutritional, socio-economic, cultural, material, and socio-psychological determinants of salt consumption for comprehensive public health campaigns. We employ an explorative scoping review of academic articles and reports, limiting our review to the original data on salt or sodium consumption published in English or French between 2000 and 2022. We describe salt consumption as research object, identifying its representation in different research fields, data sources, methodologies, samples, and links with nutritional recommendations. We synthesize existing approaches *via* four eater-oriented categories: Socio-demographic and cultural descriptors of salt consumers; Knowledge, attitudes, and beliefs on nutritional norms; Salt practices associated with dietary or medical regimes; Salt materialities: interactions and contexts. In each category, we identify the dominant relational features, i.e., what kind of ‘eater-salt’ relation is being put forward. We thus build an interdisciplinary documentary base of dietary sodium consumption factors. We discuss the results, suggesting that comprehensive nutritional policies for global salt reduction require interdisciplinary eater-oriented data frameworks.

## 1. Introduction

Salt (NaCl)^1^ and sodium (Na) are ubiquitously present in cooking and eating practices, and, at the same time, excessive sodium consumption presents serious dangers for human health worldwide. Such an ambivalence requires attention to the specificities of salt-in-action, as well as to the place of salt in the relations between food, nutrition, and health. While the World Health Organization (WHO) campaign for dietary salt reduction shapes salt and dietary sodium overconsumption as a critical public health issue, salt keeps being a necessary element for a human body (but not more than 5 g per day) (1). Salt is attributed a manifold of meanings, depending on the ways it enters into food and culinary practices (discretionary salt vs. salt hidden inside the hyper transformed products; refined salt vs. *fleur de sel*; iodized salt, etc.). Intervening into the habits of salt consumption thus requires sensitivity to its materialities (how material properties in context influence relations with salt), food routines, techniques of application (salt shakers, apps), as well as affective positions, cultural belongings, and socio-economic status of salt eaters.

In this article we propose to rethink conventional forms of representing nutritional data for intervention (graphic results of grand cohort questionnaires, such as NutriNet-Santé (2)), and, to put it more boldly, what counts for data in salt consumption. Beyond the above-mentioned ambiguities related to regulating salt consumption, our proposal is driven by Kwong et al.’s critique of the quality of measurements of salt intake in WHO European Region, based on the systematic review of the average population daily salt intake in the 53 Member States of the WHO European Region (3). We therefore propose to rethink existing approaches to the sodium reduction *via* an eater-oriented knowledge framework, categorizing heterogeneous data on salt consumption on the basis of relations and practices at the frontier of food, diet and health. Our aim is to conduct a scoping review of existing literature on dietary sodium consumption, in order to build a flexible and interdisciplinary documentary base of salt consumption factors, exposing the multiple effects of salt for human diet and health and proposing an eater-oriented format for nutritional databases. Our objectives are, first, to identify and categorize the range of disciplinary approaches, methodologies, strategies, and objectives at the crossroads of salt consumption and public health. Second, to identify specific practices and relations through which salt is known (liking, preferences, following a dietary regime). To achieve these objectives, we use scoping review, though which we build on a documentary base of dietary sodium consumption factors. Finally, we propose guidelines for further empirical research on salt consumption for comprehensive and eater-oriented nutritional policies.

## 2. Reducing salt consumption: Flexible databases for public health interventions

### 2.1. Salt consumption as public health issue

Historically, salt has provided a great service to humanity, facilitating the development of agriculture and conservation techniques (4), taxation and exchanges of goods and services (4–6), and the increase of taste values in processed foods (6). Salt and dietary sodium enhance food taste, flavor, texture, mouthfeel, and palatability; it reduces bacterial growth in meats, cheeses, and other animal products, and it also enhances hedonic attraction to vegetables among children (5, 7). Finally, iodized salt has proved to be a successful tool for iodine deficiency prevention: due to its ubiquitous use, women and infants who use iodized salt display sufficient iodine levels (8–11). Similar logic was applied to salt fluoridation (12, 13).

An excessive intake of dietary sodium and salt, however, constitutes a major challenge to public health globally, particularly touching low-and middle-income countries (3, 14–16). Analyzing the ‘Global Burden of Disease, Injuries, and Risk Factor study’ (GBD) 2013 and 2017 to identify emerging public health challenges, GBD 2013 Risk Factors Collaborators observed that diets high in sodium contribute to the risk factors for multiple noncommunicable diseases, with visible increase from 2011 onwards (17), mostly in Asian countries (18). According to the authors, the minimum risk of sodium intake approaches 5 g per day, which is also the amount recommended by the WHO. However, the current mean of salt intake in the WHO member states is almost twice the recommended value despite the agreement to reduce the intake of salt by 30% by 2025 (1, 6, 19–21). Salt overconsumption is a significant factor increasing blood pressure and leading to cardiovascular and kidney disease (6, 22). It is one of the top two dietary risk factors contributing to 1.65 million cardiovascular-related deaths each year (17, 18, 23, 24). Mostly touched by hypertension are low-and middle-income countries (25), where awareness of the dangers of salt and sodium remains low (14, 15, 26, 27).

This ambivalent position of salt in human diet (both as a necessity element and as a danger if consumed in large amounts) makes it challenging to develop effective guidelines for public health interventions. The intake of dietary sodium derives from three sources: [1] processed or manufactured foods (e.g., bread, soup, snacks, and restaurant meals); [2] salt contained in foods (e.g., celery, artichoke); and [3] discretionary salt (DS) added by consumers during cooking, food preparation and/or at the table (28). Most of the research data on salt and sodium overconsumption today concerns sodium and salt in products and processed foods (5, 29) since they are major contributors of sodium intake, particularly breads, processed meats, and sauces (30). However, these data must be accompanied by an understanding of exact consumer practices of sodium intake in order to provide a basis for effective sodium reduction campaigns. For example, Blanco-Metzler et al. (31) qualitative exploratory study in Costa Rica, where cardiovascular diseases have been the leading cause of deaths, demonstrates that most of the sodium intake daily came from domestic consumption. The authors relate this phenomenon to the beliefs that food cannot be consumed without salt, to the habits of salting generously, and the lack of awareness that processed foods already contain sodium (*ibidem*).

The midpoint of the WHO public health campaign for the decrease of dietary sodium consumption shows that low-income countries are still far from reaching an international goal (3, 32). Building on this point, we propose to revisit existing approaches to reducing salt consumption from an eater-oriented perspective, taking into account social, cultural, nutritional, interactional, and hedonic influences. Our argument also follows Kwong et al.’s warning that existing methods of measuring daily salt intake (24 h urinary collections, spot urine collection, dietary recall, food frequency questionnaires (FFQs), dietary records, household budget surveys) do not produce consistent results (3). By conducting a scoping review of existing studies at the crossroads of salt consumption and public health, we categorize studies from nutrition, health management, clinics, cultural anthropology, and sociology, thus building an interdisciplinary documentary base of sodium consumption factors. This documentary base proposes an eater-oriented framework to categorize data on salt overconsumption, linking nutritional knowledge with social sciences to facilitate heterogeneous and eater-oriented knowledge and evidence production. We suggest that databases built though this framework and grounded into the context of a particular country may provide fruitful evidence for effective public health guidelines for dietary sodium reduction in the second half of the WHO 2013 campaign aiming to decrease the amount of sodium consumed by 30% by 2025.

### 2.2. The effects of databases: Toward new guidelines for reducing salt intake

According to Durazzo and Lucarini, a better understanding of the frontier between diet and health requires new databases, especially in the perspective of factors contributing to chronic illnesses (33). This need is dictated, among others, by the search of adequate food policies, by promotion of nutritional knowledge among the consumers, and by the proven effects of balanced diet on the general health level. In this frame of reference, a collection of articles on the topic of databases and nutrition was published in Frontiers in March 2022 under the direction of Durazzo and Lucarini. This collection has presented new types of databases, dealing not only with nutritional components (for example, systematizing the types of low-or no-calorie sweeteners in products and beverages (34)), but also those linking nutritional and social characteristics to understand contemporary food scapes (for example, a database studying modernization of Malaysian food patterns (35)).

According to Leonelli, data is material artefacts which can be mobilized in a specific context of knowledge production (36). Databases thus are information infrastructures, which, according to most practitioners, should represent an open archive of knowledge for the use of many different scientific disciplines (37). According to Bowker, a “working archive” of a database is a tool for management (public health management, in our case), and it requires that “social, political, and organizational context is interwoven with statistics, classification systems and observational results in a generative fashion” (37). Since the distribution of data into the categories largely depends on the context, classification systems are doing social and political work (37).

We build on this perspective, arguing that the current state of affairs in the global campaign for dietary sodium reduction requires new flexible databases. In other words, nutritional classification of sodium consumption factors cannot be translated into an adequate policy, without taking into consideration the social, cultural, and psychological processes influencing salt consumption. We thus argue for the need of categorizing data on salt and sodium consumption around eaters’ practices and relations, rather than around disciplinary or nutritional research questions. Such an eater-oriented approach is useful not only for building comprehensive interdisciplinary health interventions, but also for rendering nutritional knowledge accessible for non-scientific audiences, thus contributing to the increase of consumers’ nutritional knowledge. Our argument resonates with Poulain et al.’s (35) observation that nutritional surveys and studies take an eating individual separately from their context, while the latter provides sociological and ethnological insights into the food habits. Leaving aside the social dimension of food consumption, however, can result in ineffective, and sometimes even contra-effective food policies and regulations. Therefore, linking nutritional knowledge with psycho-socio-cultural determinants requires new flexible knowledge frameworks.

Bowker (37) argues for flexible databases which are “as rich ontologically as the social and natural worlds they map (…)” (*ibidem*), as databases “shape the world in its image”. Through the analysis of three experimental databases, Wateron (38) has demonstrated that flexible heterogeneous databases include an exposure of the intentions, reflexivity, and policy inscribed inside them, which can be heuristic. In the case of sodium consumption, an effective database would include, at the same time, the interaction of salt with human (linking, perception learning, and body effects), its material dimensions (compositions, formats, and presentation), socio-economic issues (distributions of populations groups most at risk for sodium overconsumption), cultural influences (recipes, traditions, beliefs, and symbolic significations), common salt practices and routines, and knowledge about/attitudes toward food manufacturing and nutritional messages. Our article makes the first step in this direction, building a documentary base^2^ which would merge factors of salt consumption with eaters’ relations and practices, as well as metadata which shapes their representation (methods, units of analysis, subject framing, and geographical distribution of existing knowledge), to suggest guiding categories for salt reduction. In the following section we will expose in detail the process of our documentary base construction.

## 3. Methodology

### 3.1. Approach

We have chosen a scoping review approach to answer to our objective of rethinking salt consumption factors through an eater-oriented approach. Scoping reviews aid to explore the range, extent, and nature of existing research approaches, to summarize evidence from multiple disciplines, and to identify research gaps or openings (39, 40), which is necessary for proposing a novel eater-oriented knowledge framework. Tricco et al. (40) and Peters et al. (41) precise that scoping reviews are useful to answer broad research questions, aiming not to compare but rather to describe existing body of work on the issue and present a type of knowledge synthesis. Therefore, scoping review is consistent with our purpose of identifying the openings for eater-oriented databases on salt consumption. Through the exploratory scoping review are able to extract existing nutritional, socio-economic, cultural, material, and socio-psychological determinants and descriptors of salt consumption, synthesizing them into the eater-oriented categories, and relating them to particular methodologies, data sources, and links with nutritional recommendations. The review was conducted within the framework of a French interdisciplinary project *Sal&Mieux: Optimizing the use of discretionary salt*, however, the scope of our reviewed was broader than only discretionary salt, with interest in the overall appearance of salt in human eating practices. To watch for the review rigor, we have adopted a PRISMA extension for scoping reviews, PRISMA-ScR (40), following the best practices for scoping reviews, identified by Peters et al. (41), as well as the use of scoping reviews in food studies (42–44).

### 3.2. Search strategy and initial screening

**Figure 1:**
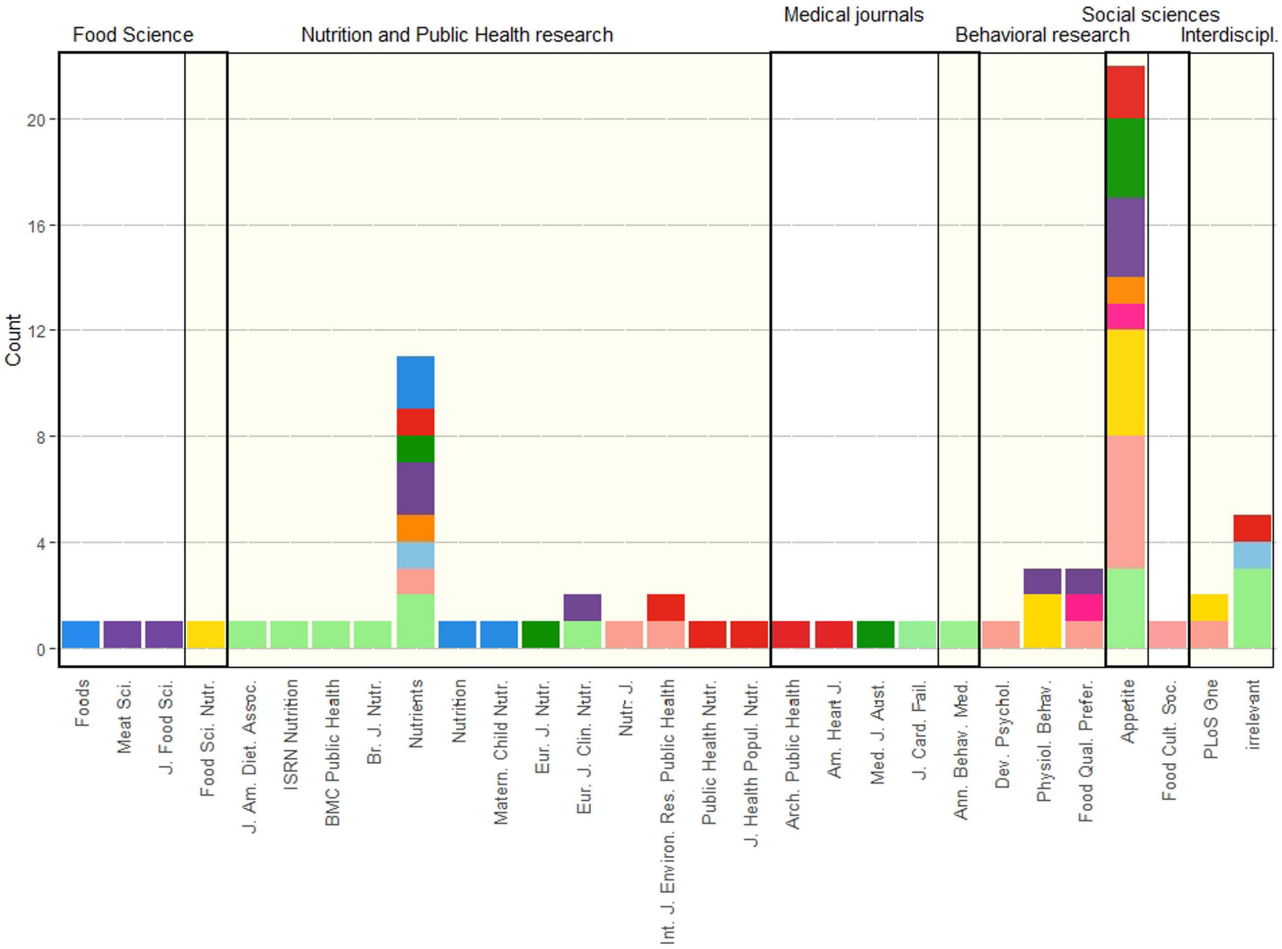
Stacked bar chart of research fields.

Our search strategy was to extract studies which articulate salt consumption and public health from eater’s perspective across disciplines: health and epidemiology, behavioral and social sciences, nutrition and public health management. The stacked bar chart of disciplinary fields and journals selected for review represents the diversity of disciplines taken into account (Figure 1). The scale of search was limited to articles in English or French^3^ published between 2000 and 2022. The search line in English was: (“Salt*” OR “Sodium”) AND “Consumption”; (“Salt*” OR “Sodium”) AND “Consumption” AND “Home”; “Salt*” AND ((“Home*” NEAR “Table” OR “Cook*)); “Discretionary salt”; (“Salt*” OR “Sodium”) AND “Consumption” AND “Health”; (“Salt*” OR “Sodium”) AND “Nutrition.” We have used several search engines: general (Google Scholar); specialized in health (ScienceDirect, NCBI PMC, PubMed); specialized in human and social sciences (Persée, CAIRN, JStore and ISIDORE). After the initial search, we have removed the duplicates and searched manually for studies appearing the reference lists of works already included. Eight criteria were used to facilitate the manual search and watch for the diversity of evidence: [1] search engines; [2] combinations of keywords; [3] publication dates; [4] publication outlets; [5] scientific disciplines; [6] research themes; [7] types of data and study designs; [8] the countries of residence of concerned population. We have finally compared our results to the existing reviews on salt and dietary sodium consumption, watching for the inclusion of the major works on the topic.

In the initial screening phase, two researchers^4^ have reviewed the titles, abstracts, and keywords from the resulting documents, coupling them with two expert interviews (with a restaurant chef and his apprentice) and assembling results into a report (45). The report was discussed by a larger scientific consortium of *Sal&Mieux* to identify eligibility criteria and objectives for the current review article^5^. The further steps, performed by three researchers^6^, consisted of returning to the full texts of the articles from the report and introducing publications from 2021 and 2022, obtained *via* the same research protocol. We have identified bibliometric data and bibliographic data of each document: authors, types of publication, publication year, DOI, numbers of quotes, summary, methodology, population, countries/territories of origin, institutions). The documents were exported to Zotero software (Zotero version: 6.0.13) for screening for eligibility and categorization.

### 3.3. Eligibility criteria and selection process

The following criteria were used for screening for eligibility: 1. original data, 2. scientific or otherwise credible data sources (such as national cohort reports), 3. citated elsewhere, 4. significant sampling size, 5. the study articulates salt consumption and public health. We have also made sure that the retained articles have not been updated by subsequent research results published by the same authors, using a similar methodology and based on a similar sample. All documents, including those left out of the review pool, have been discussed by three researchers^7^ to reach agreement on the pertinence of these works for the research objectives.

We have excluded reports from public agencies, scientific book chapters, and non-peer-reviewed articles. However, we have retained institutional reports based on nutritional epidemiological surveys in France (2, 46, 47) since they have largely informed the initial discussion within the scientific consortium of *Sal&Mieux*. Although there exists ‘gray literature’ on the use of salt by consumers (by agro-industrial companies or food distributors), we could not access it to include in our exploratory literature review. Our selection, therefore, includes only the sources accessible through academic databases.

We have retained 71 published peer-reviewed articles in French and English based on original data, published between 2000 and 2022. The older articles from medical and experimental sciences (prior to the year 2000) were also included if they marked an emergence of themes, sub-fields, and experimental designs still heavily referred to in contemporary research works (*n* = 12). Publications from the experimental sciences were selected if they provided insights into the specific salting practices and consumers’ food habits. We also retained physiological works on hedonic acceptability of salt decrease, the regulation of the appetite for salty products, and on the effects of physical activity on such appetite. The studies dealing with the influence of peers or families, as well with the expression of individual food preferences in different contexts were also retained (e.g., parental control, collective influences).

### 3.4. Categorization process

Our next step was to synthesize and categorize retained articles from an eater-oriented perspective, that is on the basis of relations and practices at the frontier of food, diet and health. The goal was to come up with new flexible categories, speaking to everyday eaters’ practices and capable to contain heterogenous multidisciplinary data.

First, we created a descriptive form to extract authors, journal type, disciplinary fields, population and sampling size, salt/sodium sources discussed, methodology and data type, and key findings for each included article. Second, this descriptive form was enriched by two *ad-hoc* columns: 1. Though what kind of relations/what practices is salt becoming an object of study (what we called “relational features”), and 2. How the study links salt consumption with nutritional recommendations. The entries in these columns were done by three authors^8^ in a collective iterative discussion, after reaching a consensus, as the data was entered into the descriptive form. Finally followed the second round of reading, structured by the relational features and links with nutritional recommendations. In this round, we have synthesized six knowledge fields, ten links with nutritional recommendations, nine relational features, and four thematic categories which describe salt consumption. This was done through a discussion and consensus among the authors. The final result of our categorization process is an eater-oriented documentary base of salt consumption factors, which is accessible in Supplementary Table 1. The following section describes this documentary base in more details.

## 4. Results

The 71 research articles coming from 29 sources^9^ were selected for this scoping review. The geography represented in our sampling included North America (United States *n =* 18; Canada *n =* 3) and South America (*n =* 6), European (*n =* 23), Asian *n =* 11), African (*n =* 8), Nordic (*n =* 3), and Oceanian countries (*n =* 10). Most of the selected articles focused on one country (*n =* 64), while five articles took a comparative perspective: Menyanu et al. (9, 15) involved samples from two countries, and four other studies based their analysis on a world-wide cross-country data collection (48–51). Two studies took samples from two countries without a comparative perspective (52, 53). The selected studies were based both on the sampling representing general population and specific population groups (those at risk, for example).

### 4.1. Eater-oriented categories to represent data on salt consumption

Five different sources of salt intake, or modalities of salt presence in eaters’ diet, were identified:

- SF: salt in raw or processed food,
- TS: table salt,
- CS: cooking salt,
- CAS: controlled added salt,
- Global: sodium consumption from all sources

To obtain an overview of daily sodium intake from the consumers, the data was mostly collected *via* dietary recalls before converting food intakes into nutrients, using standard food composition tables^10^ (*n =* 7). Other methodologies included 24-h urine collection tests^11^ (*n =* 10) or both dietary recalls and urine collection tests (54–56). One study used data modelling to assess the potential impact of reformulated products on the population salt intake (48). Out of the 39 studies that included table salt use, 21 used frequency scales (from never to always), binary yes/no questions about salt use in general or self-perceived quantities (from too little to too much) to determine salting habits. Some studies only focused on the use of table salt (TS) and cooking salt (CS; *n =* 7) (51). Some studied baby-food seasoning (57); table salt compensation (28, 58), and the use of a salt shaker (59). Six groups of authors focused on salty foodstuffs only (SF; e.g., industrial food, salty snacks, bread, spreads and dressings) (60–65).

We have extracted from the retained articles nine non-exclusive relational features, that is through what type of relations or practices does the problem of salt consumption emerge:

- Intake: concerns consumption or absorption levels during meal preparation and consumption.
- Knowledge: concerns nutritional and culinary dimensions.
- Awareness: concerns health-related risks.
- Beliefs: concerns values and representations of the benefits or harms of salt.
- Attitudes: concerns form of justification, “relation to,” “attitude toward” recommendations, prescriptions or communications.
- Behaviors/social practices: concerns routines and habits.
- Liking or Preference: concerns hedonic dimension.
- Taste perception: concerns registration of salty tastes.
- Consumer practices: purchasing salt and salt containing products.

In some instances, we have accompanied each relational feature with a more specific connotation, such as ‘caregiving practices’, ‘DS use’, ‘commercial value’.

Finally, four *ad hoc* categories synthesize existing knowledge into the dietary sodium consumption factors: [1] “Socio-demographic and cultural descriptors of salt consumers”; [2] “Knowledge, attitudes, and beliefs on nutritional norms”; [3] “Salt practices associated with dietary or medical regimes”; [4] “Salt materialities: interactions and contexts.” The categories are formulated from the articles’ keywords, titles, research questions and findings. Some formulations, such as ‘descriptors of salt consumers’ or ‘knowledge, attitudes, beliefs’ are taken directly from keywords, titles or results. Others are formulated after reading into summary by three researchers^12^. In the latter case, ‘salt materialities’ refer to the translation of salt performances and figuration of salt as both material and symbolic object configured in practice. This is inspired by Bowker and Star’s analysis of classification as a product of action and relations, creating boundaries between communities of practice (66).

The Table 1 synthesizes our corpus through the categories resulting from the scoping review. It includes [1] The four thematic categories. [2] Sampling (representative or randomized). [3] The sources of sodium intake investigated. [4] The type of data or methodology. [5] The relational features with references. The columns and rows of this table were adjusted to map the studies that have similarities to each other.

**Table 1:** Corpus synthesis through the eater-oriented categories.

*Thematic category*				*Sampling*		*Salt sources*					*Data type*				*n*=	Relational features (and article reference in the References)
Socio-demographic and cultural…	Knowledge, attitudes, and beliefs…	… dietary or medical regimes	Salt materialities…	Representative	Random	SF	TS	CS	CAS	Global	Declarative data	Objective measures	Collected data using experiments	Qualitative data		
			**X**		**X**	**X**							**X**		1	• Consumer practices, commercial values (58)
			**X**		**X**	**X**							**X**		1	• Behaviours (60)
**X**				**X**		**X**					**X**				1	• Intake (56)
	**X**				**X**	**X**					**X**				1	• Awareness, knowledge, attitude (57)
			**X**		**X**	**X**					**X**				1	• Intake (43)
**X**					**X**	**X**	**X**	**X**			**X**				1	• Liking (102)
	**X**				**X**	**X**	**X**	**X**			**X**				5	• Attitude (105, 106) • Knowledge, attitude, behaviours, beliefs (27, 44, 106)
		**X**			**X**	**X**	**X**	**X**			**X**				1	• Attitude (78)
			**X**		**X**	**X**	**X**	**X**			**X**		**X**		1	• Preference (111)
	**X**				**X**	**X**	**X**	**X**			**X**	**X**			1	• Awareness, knowledge, attitude (68)
	**X**				**X**	**X**	**X**	**X**						**X**	3	• Attitude, opinions, practices (13) • Intake, caregiving practices (70) • Knowledge, beliefs (71)
			**X**		**X**	**X**	**X**	**X**						**X**	1	• Attitude, practices (31)
		**X**			**X**	**X**	**X**	**X**				**X**		**X**	1	• Attitude, behaviours (75)
**X**				**X**		**X**	**X**	**X**			**X**				2	• Intake, awareness, knowledge, behaviours (32, 104)
	**X**			**X**		**X**	**X**	**X**			**X**				3	• Attitude (73) • Intake, beliefs, behaviours (47) • Knowledge, beliefs, attitude (45)
**X**					**X**	**X**					**X**				1	• Liking, behaviours, attitude (61)
			**X**		**X**	**X**		**X**			**X**				1	• Attitude (89)
			**X**		**X**	**X**							**X**		1	• Behaviours (59)
**X**					**X**		**X**	**X**			**X**				2	• Attitude (100) • Behaviours (46)
		**X**			**X**		**X**	**X**			**X**				1	• Intake (107)
		**X**		**X**			**X**	**X**			**X**				1	• Intake, caregiving practices (79)
			**X**		**X**		**X**	**X**						**X**	1	• Intake, caregiving practices (53)
			**X**		**X**		**X**						**X**		1	• Behaviours (54)
			**X**		**X**		**X**		**X**				**X**		2	• Behaviours, preference (28, 55)
**X**					**X**				**X**				**X**		3	• Preference (65, 66, 103)
			**X**		**X**				**X**				**X**		11	• Behaviours (88) • Preference (82, 84, 86, 108, 109, 111)• Preference, taste perception (85, 87, 110)
**X**					**X**				**X**		**X**		**X**		1	• Attitude, preference (101)
			**X**		**X**				**X**		**X**		**X**		1	• Preference (90)
		**X**			**X**					**X**	**X**				2	• Intake, attitude (77, 80)
**X**					**X**					**X**	**X**				1	• Intake (2)
**X**					**X**					**X**	**X**	**X**			1	• Intake (63)
**X**					**X**					**X**	**X**	**X**	**X**		1	• Intake, taste perception (50)
	**X**				**X**					**X**	**X**				1	• Intake, attitude, behaviours (49)
	**X**				**X**					**X**	**X**	**X**			3	• Intake, caregiving practices (69) • Intake, knowledge, attitude, behaviours (26, 51)
		**X**			**X**					**X**	**X**	**X**			1	• Awareness, attitude (48)
			**X**		**X**					**X**	**X**	**X**			2	• Intake (8, 81)
	**X**			**X**						**X**	**X**	**X**			2	• Awareness, beliefs, attitude (67, 52)
		**X**		**X**						**X**	**X**	**X**			1	• Intake, attitude (74)
**X**				**X**						**X**		**X**			1	• Intake (7)
**X**				**X**						**X**	**X**				2	• Intake (40, 62)

### 4.2. Salt consumption/health articulation

In each category, we have identified six knowledge fields which articulate salt consumption and health:

- Food Science and Nutrition;
- Nutrition and Public Health;
- Medical and Behavioral Studies;
- Behavioral Research and Social Science;
- Social Science;
- Interdisciplinary.

We have also identified ten different strategic and methodological approaches to the issue of excessive sodium consumption, which we called ‘links with nutritional recommendations’:

- Assessment of salt reduction intervention (*n* = 5).
- Awareness and knowledge as strategies for salt reduction (*n* = 9).
- Compliance with nutritional guidelines (*n* = 6).
- Consumer acceptance (*n* = 10).
- Identification of barriers to salt reduction (*n* = 2).
- Impact of material environment on salt usage (*n* = 2).
- Physiology-biological determinants (*n* = 8).
- Socio-cultural determinants and awareness and knowledge as strategies for salt reduction (*n* = 2).
- Socio-cultural determinants (*n* = 12).
- Sodium reduction (*n* = 15).

The Table 2 demonstrates the intersections between the research fields represented by specific journals, links with nutritional recommendations, and types of population concerned in the thematic category [1] “Socio-demographic and cultural descriptors of salt consumers.”

**Table 2:** Salt consumption/health articulation in the thematic category [1] “Socio-demographic and cultural descriptors of salt consumers”.

*Research fields* (*n* =)	*Populations targeted by nutritional recommendations* (*n* =)	*Links with nutritional recommendations*	*Journals* (*n* =)
**Food Science and Nutrition (4)**	*General population* (4)	Assessment of salt reduction intervention	*Foods* (1)
		Consumer acceptance	*Meat Science* (1)
		Sodium reduction	*J Food Sci* (1)
		Physiology-biological determinants	*Food Sci Nutr* (1)
**Nutrition and Public Health (25)**	*General population* (17)	Assessment of salt reduction intervention	*Nutrients* (2) *Nutrition* (1)
		Awareness and knowledge as strategies for salt reduction	*Int J Environ Res Public Health* (1) *J Health Popul Nutr* (1) *Public Health Nutr* (1) *Nutrients* (1)
		Consumer acceptance	*Eur J Clin Nutr* (1) *Nutrients* (2)
		Identification of barriers to salt reduction	*Nutrients* (1)
		Socio-cultural determinants & Awareness and knowledge as…	*Nutrients* (1)
		Socio-cultural determinants	*Int J Environ Res Public Health* (1) *Nutrients* (1) *Nutr J* (1)
		Sodium reduction	*Nutrients* (1) *Eur J Clin Nutr* (1)
	*Specific group of the population* (8)	Assessment of salt reduction intervention	*Matern Child Nutr* (1)
		Compliance with nutritional guidelines	*Eur J Nutr* (1) *Nutrients* (1)
		Sodium reduction	*J Am Diet Assoc* (1) *ISRN Nutrition* (1) *BMC Public Health* (1) *Br J Nutr* (1) *Nutrients* (1)
**Medical** **& Behavioural Studies (5)**	*General population* (1)	Compliance with nutritional guidelines	*Med J Aust* (1)
	*Specific group of the population* (4)	Awareness and knowledge as strategies for salt reduction	*Am Heart J* (1) *Arch Public Health* (1)
		Sodium reduction	*J Card Fail* (1) *Ann Behav Med* (1)
**Behavioural Research** **& Social Science (28)**	*General population* (17)	Awareness and knowledge as strategies for salt reduction	*Appetite* (2)
		Compliance with nutritional guidelines	*Appetite* (2)
		Consumer acceptance	*Appetite* (3) *Food Qual Prefer* (1) *Physiol Behav* (1)
		Identification of barriers to salt reduction	*Appetite* (1)
		Impact of material environment on salt usage	*Appetite* (1) *Food Qual Prefer* (1)
		Physiology-biological determinants	*Appetite* (2)
		Socio-cultural determinants	*Appetite* (2)
		Sodium reduction	*Appetite* (1)
	*Specific group of the population* (11)	Compliance with nutritional guidelines	*Appetite* (1)
		Physiology-biological determinants	*Appetite* (2) *Physiol Behav* (2)
		Socio-cultural determinants	*Appetite* (3) *Dev Psychol* (1)
		Sodium reduction	*Appetite* (2)
**Social Science (1)**	*Specific group of the population* (1)	Social and cultural determinants	*Food Cult Soc* (1)
**Interdisciplinary (7)**	*General population* (2)	Awareness and knowledge as strategies for salt reduction	Irrelevant (1)
		Socio-cultural determinants & Awareness and knowledge as…	Irrelevant (1)
	*Specific group of the population* (5)	Physiology-biological determinants	*Plos One* (1)
		Socio-cultural determinants	*Plos One* (1)
		Sodium reduction	Irrelevant (3)

### 4.3. Salt and dietary sodium consumption factors as guidelines for salt reduction

In this section we will briefly discuss each thematic category of salt consumption factors, providing an overview of existing knowledge. As the aim of this article is a scoping review to propose an interdisciplinary and eater-oriented knowledge framework, we will not discuss each article individually. The findings of the individual articles can be found in the documentary base presented in the Supplementary Table 1.

#### 4.3.1. Socio-demographic and cultural descriptors of salt consumers

Eighteen works have been classified in this category. Ten of them were national demographic studies, and six were based on population-representative samples. Nine studies used declarative data to measure the overall salt intake or specific salty food consumption (e.g., snacks) among particular population. Four institutional reports presented declarative data obtained from the representative samples of French population (2, 46, 47, 67). None of these national surveys used 24-h urine collection as methodology. Studies using objective measures were rare in this first category. Some of them aimed to evaluate compliance with nutritional guidelines: Piovesana, Sampaio, and Gallani, for example, focused on a random sample of 108 hypertensive and normotensive participants (54). Iacone et al. in their study of the influence of iodized salt consumption on the iodine levels among the pediatric population evaluated compliance with nutritional guidelines regarding sodium and iodine (8). The study of Huggins et al. (68) characterized table salt consumption with a cohort sample of 784 Australians.

Five articles were based on data from controlled experiments focused on the preferences for salt. Two articles evaluated gustatory perception thresholds, highlighting socio-demographic, cultural, and biological descriptors of salt preferences and perception patterns (54, 69). Beauchamp and Cowart (70), meanwhile, treated salt consumption in perspective with socio-economic and racial characteristics. Finally, this category also contains articles presenting cultural descriptions of salt preferences. Kerrihard et al.’s (71) work, for example, explored the effect of acclimation in the United States on hedonic evaluation of salt. Drewnowski et al. (69) focused on the relationship between age, gender, perception of salt taste, and actual sodium consumption.

#### 4.3.2. Knowledge, attitudes, and beliefs on nutritional norms

This category includes 21 works. Like in the previous category, declarative data was used in studies relying on questionnaires or food records (*n =* 14). Six publications combined questionnaires and objective measures (24-h urinary sodium excretion). Four works were based on the qualitative material obtained through the semi-structured interviews and focus groups; one relied on intervention to reduce added salt during cooking (56).

The choice to include knowledge, attitudes, and beliefs in the same category was consistent with the combination of keywords chosen by the articles’ authors (15, 49, 55, 56, 61, 72). However, some works demonstrate the relevance to distinguish the roles of knowledge, attitudes, and beliefs, since practices and recommendations in the three cases may differ. In some cases, beliefs and knowledge linked to chronic illnesses have been studied to investigate how knowledge structures diet in diabetes management (73). Other studies treated the transmission of knowledge and everyday skills from dieticians as compared to the self-help literature for people with hypertension (74). Rhodes et al. (64), also addressed the transmission of knowledge within the multicultural and intergenerational families. Family, therefore, can be considered as an institution for socialization to salt consumption (75, 76).

The barriers to salt reduction among the general population also appear in this category (*n =* 5), often pointing to knowledge about the official dietary sodium recommendations (50). Another mention is the difficulty of differentiating between sodium and salt, as well as calculating the ratio of one to another (61, 77). Other issues addressed in this category include knowledge about the sources of sodium intake (*n =* 8), awareness about the salt-related health risks (*n =* 10), beliefs about the nutritional or symbolic values of certain salts (e.g., sea salt, iodized salt) (52, 56).

This category, finally, includes socio-psychological factors which play a role in salt consumption, reinforcing Ahn et al.’s (78) invitation for a tailored intervention approach based on case-by-case public health campaigns and addressing different stages of behavioral change. Outcome expectancy, barriers, knowledge, purchasing skills also deserve to be seen as marketing issues, as salt reduction is best achieved and maintained with the concrete goals and rewards (79).

#### 4.3.3. Salt practices associated with dietary or medical regimes

This thematic category includes eight works on adherence and attitudes toward nutritional and medical guidelines, as well as on the impact of some dietary regimes on salt intake. All records in this category used reported data collection in the form of questionnaires and diet recalls. Four studies went further, measuring excreted sodium from participant’s urine or comparing declarative data to the data obtained *via* objective biomedical measurements. In these cases, objective measures allowed: (a) to evaluate the effects of a multi-faceted and population-wide salt reduction intervention (80); (b) to assess the impact of a controlled experiment where healthy adults would follow an appropriate amount of daily salt intakes (81); (c) to assess the impact of a controlled experiment where people with heart failure would follow an appropriate low-sodium diet (53); (d) to assess the role of the absence of the table salt or the use of salt substitutes on hypertension and stroke (82). Articles by Chung and collaborators, as well as those by Adriouch et al. and Henson et al. (53, 83, 84), put salt consumption in perspective with cardiometabolic diseases, arguing for the necessity of a particular dietary regime. We have finally included in this category the articles on dietary regimes: Bournez et al.’s (85) work on children’s dietary regimes; Dyett et al.’s (86) paper on the effect of daily vegan diet on sodium and other nutrients’ intake.

#### 4.3.4. Salt materialities: Interactions and contexts

Our fourth category assembles studies (*n =* 24) which regard sodium as belonging to or interacting with different matters, including the influence of the environment. The predominant methodology here is experimental protocols (*n =* 18); some have relatively small study samples (participants are less than 83 in 55% of cases). Experiments generally rely on pre-salted preparations or solutions (called CAS for “controlled added salt”; *n =* 16). A significant proportion of reviewed articles (*n =* 12) deal with the physiological processes related to salt consumption and iodine intake (9, 87). The study of Frye and Demolar (88), for example, attempted to relate sodium intake to women’s menstrual cycle; however, the results did not reveal a dependency. A number of works dealt with the physico-chemical composition of the meal, physiological interactions, and preferences (89, 90); with the table salt compensation strategies (59) or personal acceptability of salt reduction (91).

The second common feature is that the articles in this category pay attention to how material elements or specific environment influence perception and preferences (31, 92, 93), behaviors (58, 94), attitudes (95), caregiving (57), and consumer practices (62, 63). This is an important relational factor in understanding salt consumption. Materialities identified in this category also include variations of the widths of holes in salt shakers (58); noise variations during tasting (94); the influence of summer heat on sodium loss and salt avidity (96), and low-salt food alternatives (63). Finally, one article in this category approached salt materialities through data modelling methodology (48). The authors conducted dietary impact modelling to demonstrate that product reformulation by the food industry has the potential to contribute substantially to salt-intake reduction without jeopardizing products’ taste values, yet this process should be supported by a multi-stakeholder approach (48).

## 5. Discussion and further research directions

Salt and its excessive intake have been approached from manyfold research positions: scholarly works on salt reduction can be found in the fields of nutrition and food sciences, public health and health management, biomedical and clinical sciences, cultural anthropology, social and behavioral sciences, and interdisciplinary works. In this section, we discuss the results of our scoping review through the lens of other reviews conducted on the subject of salt consumption, identifying the dominant approaches and the novelty of our eater-oriented knowledge framework for building effective public health interventions.

The reviews of salt consumption and reduction can be divided into three approaches. The first is an *evidence-providing approach* for regulating high rates of salt consumption. The examples of such approach are: Moschonis and Karatzi’s (97) study of dietary approaches for decreasing hypertension (the major factor contributing to cardiovascular diseases); Campbell and Train’s (98) argument for labelling the dangers of salt on the packages and shakers; Wong et al.’s (21) assessment of the studies on dietary salt in relation to health outcomes. This approach is also present is the systematic reviews of interventionist scientific studies. For instance, Tsirimiagkou et al. (99) reviewed the scientific literature on the relationships between sodium intake and three cardiovascular diseases (arteriosclerosis, arterial remodeling and atheromatosis), concluding that the issue requires further interventional scientific studies.

The second approach is *managerial approach to salt reduction*. Through this approach, He et al. (6) reviewed different strategies of salt consumption management. The authors also evoked Asaria et al.’s (100) argument of cost-effectiveness of salt intake decrease for combatting the epidemics of cardiovascular diseases in the developing countries, where salt intake is extremely high. Jaenke et al. (101) have taken a further step in researching salt overconsumption management, conducting a systematic literature review to understand how products can be reformulated for lesser salt without losing consumer acceptability. Their results have shown that a < 40% salt reduction in breads and approximately 70% in processed meats (obtained as a result of sodium compensation and/or replacement) would not significantly impact consumer acceptability. Some other examples of a managerial approach to reviews on salt overconsumption include Regan et al.’s (29) review of the current reformulation strategies in regard to consumer behavior or Eyles et al.’s (19) study on the use of smartphone apps for salt reduction.

Finally, some reviews on salt reduction take a *nutri-behavioural, socio-demographic and cultural approach* to the problem of salt overconsumption. Darmon and Drewnowski’s review, for example, associates higher sodium intake with lower socioeconomic status as the latter supposes lower quality diet due to the limited economic resources (102). In another study, Laisney (103) noted that teenagers from lower socio-economic backgrounds are especially at risk for salt consumption. This approach also reveals regional specificities of sodium intake.

In this article, we aimed for the development of a new, *eater-oriented approach* at the intersection of salt consumption and public health. This approach challenges the boundaries between the different communities of practice (nutritional science, sociology, public health, etc.) and assembles heterogeneous knowledge on salt intake around the figure of an eater. Such an approach serves as a model for flexible and eater-oriented databases on dietary sodium consumption for effective public health interventions. Our eater-oriented categories synthesize a wide array of relational dynamics involved in salt consumption: socio-demographic, methodological, contextual, technical and technological, affective, communicative, and deliberative. These dynamics can translate as interdisciplinary guidelines for decreasing salt intake.

We have identified four non-exclusive thematic categories which help to understand salt consumption: [1] Socio-demographic and cultural descriptors of salt consumers; [2] Knowledge, attitudes, and beliefs on nutritional norms; [3] Salt practices associated with dietary or medical regimes; [4] Salt materialities: interactions and contexts. Each thematic category gravitates toward particular relational feature: the second block deals mostly with attitudes, beliefs, knowledge; the first block concerns mostly intake and preference. The third category presents a wide relational scale, from caregiving practices to intake and preferences. Finally, salt materialities are associated mostly with preferences and taste perceptions. We propose that the variety of salt sources, techniques, and technologies of salting, as well as different modalities of sodium appearances in the human diet should be further researched. Taking salt materialities and interactions seriously is important for understanding compensation strategies during salt reduction programs (48, 59) and also for a coherent coexistence of different public health initiatives. The latter point has been raised by Iacone et al. and Menyanu et al. (8, 9) in relation with the usage of iodized salt for preventing iodine deficiencies.

Our scoping review shows that while there are numerous reports relying on declarative or experimental data (column G in the Supplementary Table 1), there is little qualitative data on the practices of salt consumption. Most studies in our review drew data from dietary recalls or 24-h urine collection tests. However, Blanco-Metzler et al.’s (31) explorative qualitative study of the food practices and perceptions related to excessive consumption of salt/sodium in Costa Rica shows the benefit of approaching salt overconsumption as a complex phenomenon across the different thematic categories. Blanco-Metzler et al. relied on ethnography and ethnology to understand salt-related practices from the participants’ perspective, watching out at the same time for different environmental contexts (different regions, eating out/at home), different socio-demographic and cultural profiles (age, sex, cultural roots, etc.), and individual knowledge, beliefs, and perceptions. We propose that future works employ cross-thematic qualitative approach from eaters’ perspective, and we argue that our categorization system can be used as framework for conducting qualitative interviews and observations. The documentary base presented in this article (Supplementary Table 1) can serve as a guide for this endeavor.

## 6. Conclusion

In this article, we argue that in order to achieve the WHO goal for decreasing the amount of dietary sodium consumed by 30% by 2025 there is a need for interdisciplinary and eater-oriented knowledge framework, which would include nutritional knowledge, as well as the dominant beliefs, attitudes, and practices of salt consumption. This knowledge framework would serve as guidelines for building flexible databases, informing public health campaigns. As health-and-diet databases are performative, that is to say that they both emerge from and perpetuate certain practices (38, 104), they have a considerable implication for the social and natural orders. For example, a relational database of rare diseases in France (assembling knowledge across communities of patients, health practitioners, and institutions) have challenged the production of knowledge on rare diseases (105). New eater-related databases of salt and sodium consumption, we argue, may not only inform more effective public health measures, but also make a subject more accessible for the general population.

We therefore have built an eater-oriented documentary base (available in the Supplementary Table 1), which would serve as knowledge framework for further databasing the factors of dietary sodium consumption. For this, we conducted a scoping review of existing academic literature on dietary sodium consumption, following a PRISMA-ScR checklist and best practices for scoping reviews (40, 41). We have selected 71 studies published in English and French and falling into the nexus of salt consumption and public health for a detailed review and categorization. The selected works presented a heterogeneous pool of disciplines, methodologies, geographies, salt sources, population samples, and data types. Through the two steps of categorization, we have designed a knowledge database around eater-oriented interdisciplinary categories: [1] Socio-demographic and cultural descriptors of salt consumers; [2] Knowledge, attitudes, and beliefs on nutritional norms; [3] Salt practices associated with dietary or medical regimes; [4] Salt materialities: interactions and contexts. We have also categorized each article according to the dominant relational features (how salt becomes an issue): Intake; Knowledge; Awareness; Beliefs; Attitudes; Behaviors/social practices; Liking or Preference; Taste perception; Consumer practices. Finally, we have extracted ten different strategic and methodological approaches to the issue of excessive sodium consumption, which we called ‘links with nutritional recommendations’. The synthesis of the resulted knowledge framework is presented in the Table 1, and the full documentary base—in the Supplementary Table 1. This documentary base, we argue, can serve as a framework to classify empirical and contextualized data in order to design an adequate public health response to the issue of dietary sodium consumption. Following this perspective, we have proposed guidelines for further research on salt and sodium consumption, as well as for effective public health interventions. These guidelines accentuate the contexts of food intake, eaters’ knowledge, habits and practices, cultural predispositions, meal preparation routines, and consumption environment.

## Author contributions

AE: conceptualization, data collection, investigation, analysis, writing—original draft, and writing—review and editing. PC (equal contribution with AE): data collection, investigation, software, visualization, writing—original draft, and writing—review and editing. TT-D: funding acquisition, project administration, and writing—review and editing. J-PP: writing—review and editing and resources. LT: writing—review and editing. AD: project administration, investigation, methodology, writing—original draft, writing—review and editing, and supervision. All authors contributed to the article and approved the submitted version.

## Funding

This work was supported by the French National Research Agency (ANR) as part of the Sal&Mieux project under grant (ANR-19-CE21-0009).

## Conflict of interest

The authors declare that the research was conducted in the absence of any commercial or financial relationships that could be construed as a potential conflict of interest.

## Publisher’s note

All claims expressed in this article are solely those of the authors and do not necessarily represent those of their affiliated organizations, or those of the publisher, the editors and the reviewers. Any product that may be evaluated in this article, or claim that may be made by its manufacturer, is not guaranteed or endorsed by the publisher.

## Footnotes

^1^In this article we will principally use ‘salt’ as a signifier for both salt and dietary sodium.

^2^Presented in Supplementary Table 1.

^3^The choice of languages is dictated by the authors’ language capacities. Restricting the search to the works in French and English may limit the findings of this article, while opening a path for further research in other language frameworks.

^4^PC (100%) and AD (50%).

^5^PC, AD, TT-D, LT, J-PP, AE.

^6^AE, PC, and AD.

^7^AE, PC, and AD.

^8^AE, PC, and AD.

^9^Sources other than scientific journals are labelled "irrelevant" [scientific book chapters (*n =* 2), institutional reports (*n =* 4)].

^10^ANSES-CIQUAL food composition table (*n =* 1); Arnault et al.’s "Table de composition des aliments NutriNet-Santé" (2013) (*n =* 3); Nutrition Data Systems for Research software version 5.0_35 (*n =* 1); Ethiopian and Tanzanian food composition tables (*n =* 1); United States, United Kingdom and Dutch food composition table (*n =* 1); unknown conversion method (55,566).

^11^In this section whenever the number of articles is 3 or more, we do not repeat authors’ names leaving just the number of articles. The exact names can be found in the Supplementary Table 1.

^12^PC, AD, and AE.
