# Supplementary material for: Eater-oriented knowledge framework for reducing salt and dietary sodium intake (scoping review)
Source: Front Nutr. 2023 Feb 28;10:1110446. doi: 10.3389/fnut.2023.1110446 (PMC10011657; doi:10.3389/fnut.2023.1110446)
Supplement: Supplementary file 1 [file Data_Sheet_1.PDF]

Supplementary Material

Eater-oriented knowledge framework for reducing salt and dietary sodium intake (scoping review)

Alexandra Endaltseva[*†], Paul Coeurquetin[†], Thierry Thomas-Danguin, Jean-Pierre Poulain, Tibère Laurence, Anne Dupuy

*** Correspondence:** Alexandra Endaltseva: alexandra.endaltseva@univ-tlse2.fr

Table 1. Eater-oriented documentary base of salt consumption factors

Thematic category	Reference from A to Z	Population/Sample	Salt as object (Aim/ → **Relational feature**)	Links with nutritional recommendations	Salt sources	Methodology	Key findings/Comments
**Socio-demographic and cultural descriptors of salt consumers**							
	(106) Antunez et al., 2022	URY<br><br>*n* = 498<br><br>Non-probabilistic sample recruited via two social networks; 73% women	To explore the effect of sociodemographic and psychographic characteristics on the use of discretionary salt<br><br>→ **Attitude**	Psycho-social determinants of the discretionary salt (DS) usage (time orientation and subjective risk perception of sodium consumption)	TS, CS	**Declarative data:** “Consideration of future consequences «scale survey: choosing from bread packaging, a series of statements measuring perceived risk of sodium consumption ,and a short questionnaire to collect socio demographic data.	Age is the most important predictor of discretionary salt usage: the older population is less likely to add salt. Gender presented a low contribution to discretionary salt usage. People with a higher tendency to consider immediate consequences of their eating behaviour were less likely to add salt when eating and or cooking, both at home and when eating out. The likelihood of using DS was higher for those showing lower subjective perception of severity and higher perception of risk compensation (i.e. by being engaged in other behaviours that contribute to reducing potential hazards).

	(46)<br>ANSES, 2017	FRA<br><br>*n* = 4095<br><br>A representative sample of the French population in three cohorts: 0-10- and 11–17-year-olds, adults up to 79	To measure general salt consumption among the French population; to determine the primary dietary sources of salt; to compare these data between social groups (based on age, sex, education level)<br><br>→ **Intake**	Sodium reduction among specific sub-groups of the population. The data is then used to update the PNNS advertising campaign	Global	**Declarative data:** 24-h dietary recall with self-reported DS intake	The average daily salt intake is 4,4g for children, 6,5 g for teenagers, 8,0 g for adults.<br><br>The condiments, herbs, spices, and dressings (including DS), account for 13% and 12% of sodium intakes among 0–17-year-olds and adults, respectively.<br><br>There is no significant difference between social groups regarding sex and education level
	(71)<br>Beauchamp and Cowart, 1990	USA<br><br>Two experiments<br><br>N1 = 28 Black children<br><br>N2 = 58 Black and White children and 30 of their parents. Children and children-parents' dyads were recruited at the Well Baby Clinic of Pennsylvania Hospital	To measure preferences for salt concentration among children and to draw a comparison between Black and White children, as well as between children and their parents<br><br>→ **Preference**	Social and racial determinants of salt and consumption	CAS	**Collected data using experiments:** vegetable soup prepared with 4 different concentrations of salt under controlled conditions. Each subject was presented twice with a possible pair of stimuli, after which they were asked to indicate which one was preferred	Children prefer higher sodium concentrations than their parents. No difference between the two sub-samples of Black and White children was identified.
	(70)<br>Drewnowski et al., 1996	USA<br><br>*n* = 48<br><br>Young adults aged 20 to 30 years (*n* = 24) and 24 older adults aged 60 to 75 years (*n* = 24); recruited through advertising in Ann Arbor, Michigan; both sub-samples included 50% of women	To study between ageing, perception of salt taste, and sodium consumption due to age and gender.<br><br>→ **Taste perception, preference**	Sodium reduction among specific sub-groups of the population	CAS, SF, TS, CS	**Collected data using experiments:** subjects tasted and rated five sodium solutions<br><br>**Declarative data:** food intakes were assessed using a 24-h recall and a 14-days diet record in order to examine the link between salt taste perception and preferences, as well as actual sodium intakes as a function of age and gender	Both adults and seniors were equally able to identify and rank chicken broth by their sodium concentration. The elderly participants did not have problems with salt taste perception.<br><br>The hedonic perception of the different concentrations did not differ, although the elderly was more attracted to less salted solutions.<br><br>Both salt perception and salt liking were weakly correlated with age.

	(62)<br>Dunford, Poti, and Popkin 2017	USA<br><br>*n* = 123,225<br><br>A representative sample of US population in 2 cohorts: 50,052 children aged 2-18 years and 73,173 adults aged 19; between 1977 and 2014	To examine short- and long-term trends in the contribution of snack food sources to dietary sodium intake for US adults and children from 1977 to 2014. Patterns of snack food consumption, trends in sodium intake from snack food, trends in food and beverage sources of sodium across race-ethnic, age, gender, body-mass index, household education, and income groups.<br><br>→ **Intake**	Sodium reduction among the general population. Social and racial determinants of sodium preference and consumption	SF	**Declarative data:** The 'Snack' category within the 8 national surveys included eating occasions defined by the participants as 'snack' and those related to snacking (food/beverage break). Each eating occasion was self-defined: respondents were asked to name the type of each eating occasion, and the main meal planner was asked to declare data concerning children under the age of 12. Snacking trends were studied by dividing population into age groups, BMI categories, race-ethnic groups, income groups using the Federal Poverty Level, and household education groups.	In all socio-demographic subgroups, there was a significant increase in per capita sodium intake and the proportion of sodium intake derived from snacks from 1977–1978 to 2011–2014 ($p < 0.01$). Those with the lowest household education, Non-Hispanic Black race-ethnicity, and the lowest income had the largest increase in sodium intake from snacks. While in 1977–1978, Non-Hispanic Blacks had a lower sodium intake from snacks than Non-Hispanic Whites ($p < 0.01$), in 2011–2014, they had a significantly higher intake.

	(68)<br>Esteban, 2018	FRA<br><br>*n* = 2834; 1279<br><br>A representative sample of adults and young people (aged 6-17)	To measure overall salt consumption among the French population; to determine the main dietary sources of salt; to compare these data between social groups (based on age, sex, education level)<br><br>→ **Intake**	Sodium reduction among specific sub-groups of the population	Global	**Declarative data:** 24-h dietary recall for food-based salt intake calculation and self-reported DS intakes	Men consume more salt than women. 30% of women consume less than 6 g of salt per day, compared to 11.9% of men. One-third of men can be considered as high salt consumers, with 32,1% consuming 10 g or more daily. For both women and men, the proportion of high salt consumers has increased in the space of 10 years.<br><br>In terms of the level of education, there is no link with consumption among men. On the other hand, more women who consumed less than 6 g per day reported a level of education below the baccalaureate. A decrease in compliance with health recommendations tends to be greater among women with higher education levels.<br><br>These gender differences can also be observed among children and adolescents (50.4% of girls and 29.7% of boys were part of the 40.1% consuming less than 7 g per day in the age group 6-17.<br><br>10,8% of boys can be considered as high salt consumers with an intake of 10 g or more per day. Similar contrast was observed among adolescents (15-17 years), with 26.9% of young men compared to 7.9% of young women reporting salt overconsumption.

	(107) Guàrdia et al. 2006	ESP<br><br>*n* = 392; 98<br><br>Consumers from a random sample of Catalan IRTA's panel including 195 women; ninety-eight other consumers were recruited as an untrained sensory panel, including 54 women	To compare declared salt attitudes between men and women and to evaluate gender-related differences concerning salt preference, as well as smoking habits and education level<br><br>→ **Attitude, preference**	Consumer acceptance of salt reduction	CAS	**Declarative data:** self-administered 45 items survey<br><br>**Collected data using experiments: acceptability** evaluation (scoring scale) of seven different fermented sausages prepared under controlled condition	Women had a more positive attitude than men towards reformulated products; women were more capable of sensing the differences between sat-reformulated sausages than men.<br><br>Tobacco use appears to affect beliefs about the health effects of salt. Non-smokers tended to be more favourable to a reduction of salt as part of a healthier diet.<br><br>The level of education was linked to more sensitivity to the subjective norms and social desirability effect about salt consumption.
	(69) Huggins et al., 2011	AUS<br><br>*n* = 784<br><br>Participants from Melbourne Collaborative cohort; 52% women	To characterise the population's dietary salt consumption. To assess sodium intakes through a questionnaire and a 24-h urine collection and test<br><br>→ **Intake**	Compliance with nutritional guidelines	Global	**Declarative data: questionnaire** about dietary intake and the use of discretionary salt<br><br>**Objective measures:** 24-h urinary sodium excretion	Only 1.6% of men and 7.8% of women reached the recommended salt intake level: <70 mmol sodium in urine or 4 grams of salt per day.<br><br>Those who reported salting their dishes when cooking had a 19% higher Na excretion rate than those who reported not doing so. No difference was observed regarding table salt.

	(7)<br>Iacone et al., 2021	ITL<br>*n* = 1270<br>Nationwide sample of children and adolescents (6-17 years of age); recruited with the collaboration of the Italian SIGENP	To assess sodium and iodine intakes through a 24-h urine collection and to evaluate adequacy with the EFSA (for iodine) and the WHO (for sodium) recommendations.<br><br>→ **Intake**	Compliance with nutritional guidelines	Global	**Objective measures:** 24-h urinary sodium excretion	Consumption of salt and iodine were found to be linked. When the population was divided into quartiles based on salt consumption, a gradual increase in iodine intake was seen from the first to the fourth quartile. Only participants in the top quartile of salt consumption fulfilled the EFSA sufficiency criterion for iodine intake in all age groups.<br><br>According to estimates, the quantity of iodine delivered by iodized salt is lower than the amount provided by diet in all age and sex groups. This amount is also higher for male teenagers because of their greater salt consumption. Regardless of age category, most individuals who followed the WHO recommendations of a total salt intake of less than 5 g/day did not get enough iodine.
	(72)<br>Kerrihard et al., 2017	USA<br><br>*n* = 100<br><br>Random sample of participants in two cohorts: 50% subjects presently living in the United States for more than 2 years continuously, 50% subjects that have lived in the USA for less than two years; recruited via emails, flyers, and social media; 46% are women; mean age is 23.8 (±4.46)	To examine the effect of acclimatisation to the United States on responses to salt levels in foods<br><br>→ **Preference**	Cultural determinants of salt preference and consumption	CAS	**Collected data using experiments:** hedonic response evaluation (positive response metric) of five differently salted mashed potatoes prepared under controlled conditions. Data was retrieved by FaceReader technology	The results show an association between living in the United States for an extended time and an increased positive response to foods with higher salt content. This result is explained by the fact that a prolonged period of high salt consumption leads to a greater preference for such sodium levels in food.<br><br>However, no significant difference between genders was observed.

	(108) Lampuré et al., 2014	FRA<br><br>*n* = 37,181<br><br>A random sample of the French population of the NutriNet-Santé cohort; 43,40% women	To compare the liking of sweet-fat and salt-fat sensation to age, sex, dieting, BMI, smoking, alcohol drinking, and occupational category<br><br>→ **Liking**	Sodium reduction among specific sub-groups of the population	SF, TS, CS	**Declarative data:** self-administered online questionnaire. Thirty-one items concerned the liking of fat-salt sensation	In men, age was negatively associated with a strong liking for the fat-and-salt sensation.<br><br>Women who were currently dieting were more likely to strongly like salt-fat and sweet-fat sensations than women who had dieted in the past that were less likely to be attracted to salty fat.<br><br>This positive association was also observed for obese individuals.<br><br>Men, manual workers and employees, smokers and alcohol heavy drinkers had higher liking scores for the fat-and-salt sensation than women, non-smokers, non-alcohol drinkers, or managerial staff
	(109) Mennella et al., 2014	USA<br><br>*n* = 108<br><br>A random sample of children (5-10 years of age; 61 singletons; 19 sibling pairs; 3 sibling triads) and 83 of their mothers (treated as adult population, e.g., without accounting for relational aspect)	To compare salt preference of children and adults<br><br>→ **Preference**	Biological and demographic determinants of salt preferences	CAS	**Collected data using experiments:** five soup stimuli with varying levels of sodium were used to determine the most preferred level of saltiness. Children and mothers had to taste two paired items for 5 seconds and then point to which they liked better, without instruction on how the stimuli differed. This pattern continued until the participant either chose two consecutive times the same concentration paired with both a higher and lower concentration, or chose the highest or lowest concentration twice	For meat broths, children prefer higher salt concentrations than their parents

	(2)<br>NutriNet-Santé, 2010	FRA<br><br>*n* = 159,803<br><br>A random sample of French adults of the NutriNet-Santé cohort; 75.9% women; 47.7% over 45 years old and 9.7% under 25; 60.1% employed and 15.7% retired or pre-retired	To measure overall salt consumption of the French population; to determine the primary dietary sources of salt; to compare these data between social groups (based on age, sex, education level)<br><br>→ **Intake**	Sodium reduction among specific sub-groups of the population	Global	**Declarative data:** self-administered survey	Average daily intakes are higher among men (9.2 g) than women (7.6 g). Thirty-six of women and 67% of men have salt intakes above the maximum threshold that France set to achieve in 2008 (8 g per day).<br><br>Three quarters (6.3 g/d) of salt comes from the food consumed, and one quarter is added by the consumer, either when cooking food or directly in the dish.<br><br>No difference in salt intake was observed in relation to household income.<br><br>Salt intakes are higher for overweight people (25<BMI≤30) and lower for those with lesser BMI regardless of caloric intake.<br><br>The lowest levels of sodium intake are observed in the 18-25 and over 65 age groups.<br><br>Salt intakes are higher among farmers of both genders. The lowest intakes are among retirees.

	(56) Piovesana, Sampaio, and Gallani, 2013	BRA<br><br>*n* = 108<br><br>A random sample of participants (54 hypertensive and 54 normotensive people); recruited at outpatient clinics specialised in hypertension in Southeast Brazil; 85% female; mean age was 46.04 (±10.8)	To measure the gustatory threshold for salt and its relationship with dietary salt intake among hypertensive and normotensive study participants<br><br>→ **Intake, taste perception**	Sodium reduction among people with risk factors	Global	The study design includes<br><br>**Declarative data:** a questionnaire (on discretionary salt use and a 24-h recall of dietary intake).<br><br>**Objective measures:** a 24-h urinary sodium excretion collection; and<br><br>**Collected data using experiments:** an organoleptic test to assess the threshold for detecting sodium chloride present in different proportions in water. For the latter element, the lowest detection threshold against which the person was able to perceive salt on three occasions was considered to be their detection threshold	Hypertensive subjects had a higher detection threshold than normotensive. Hypertensive subjects started to detect NaCl thresholds when these became high.<br><br>Salt intakes (collected with questionnaires and objectified with excretion analysis) were higher among the hypertensive individuals than the normotensive study participants.

	(110)<br>Purdy and Armstrong, 2007	GBR<br><br>*n* = 360<br><br>Representative sample (based on socio-economic status) of Northern Irish; 78.3% women	To evaluate discretionary salt intake and processed foods consumption, as well as consumers' awareness of the associated health risks<br><br>→ **Intake, awareness, behaviours**	Cultural determinants of salt preference and consumption<br><br>Awareness, knowledge, and strategies for salt consumption and reduction among the general population	SF, TS, CS	**Declarative data:** questionnaires at the entrance to retail food outlets and in shopping areas. The questions included dietary salt intake from discretionary salt and processed food, as well as consumer knowledge of the amount of salt in processed foods, consumer awareness of the health risks associated with high salt intakes.	Consumers reported using salt occasionally at the table (43.1%) and when cooking (35.8%).<br><br>Men reported higher discretionary salt use than women. Men were also (63%) more likely to frequently or always use salt at the table, with generous shake and heavy covering.<br><br>No significant differences between men and women were observed regarding cooking salt.<br><br>The age groups 20-29 years and 40-49 years were the heaviest consumers. The lowest socio-economic statuses had higher salt consumption than the others, but the results were not significant about discretionary salt specifically. The authors state that it is mainly ready-made meals that account for sodium intake in these cases.

	(32)<br>Santos et al., 2021	AUS<br><br>*n* = 1217<br><br>A nationally representative sample of Australian adults aged 18 years; recruited by a random digit dialling (landlines and mobile lines)	To determine if there were socio-demographic differences in salt behaviours<br><br>→ **Intake, knowledge, attitude, behaviours**	Socio-demographic determinants of salt consumption and behaviours	SF, TS, CS	**Declarative data:** telephone interviewing survey consisting of 160 questions including socio-demographic and health-related questions. Four questions dealt with salt-related aspects: identified processed foods as the main source of dietary salt; perceived salt consumption; habits of using cooking salt; table salt; and declaration of intention to cut down on salt consumption	Eighty-five percent of participants knew that processed foods are the main source of salt in the Australian diet. Almost 60% of participants frequently added salt during cooking/ meal preparation and 42% of respondents frequently placed a salt shaker on the table during meals. No consistent pattern between salt-related knowledge, attitudes and beliefs, and socio-demographic factors were found. Males, younger, and overweight/obese participants perceived their salt intake to be more than recommended.

	(67)<br>Ton Nu, MacLeod and Barthelemy, 1996	FRA<br><br>*n* = 222<br><br>A random sample of young French people aged 10-20; recruited by their parents (working at Nestlé France S. A.) or by their teachers at a school in Montpellier; 128 girls; 136 people had reached puberty	To assess gender and age-related differences between French children and teenagers in terms of food preference and habits<br><br>→ **Liking, behaviours, attitude**	Social and physiological determinants of salt preference	SF	**Declarative data**: self-administered questionnaire including 9 items relating to food preferences	Salt liking increases gradually between the 10th and 20th year, and it remains the most appreciated taste sensation before sweetness. There are no gender differences in this evolution.<br><br>For the majority, the age of 10 years old is the time of dietary change. The increase in snack consumption among adolescent males occurred at 15.8 years and concerned 3.6% of the 222 participants.<br><br>In contrast, young adolescent girls tend to become more preoccupied with their bodies and weight than boys, which leads to a decrease in their consumption of sweet foods in particular.
	(52)<br>Wardle et al., 2004	Twenty-three countries including FRA<br><br>*n* = 19,298<br><br>University students aged 17 to 30; 10,816 women	To assess gender differences in health behaviours among an international sample of young adults<br><br>→ **Behaviours**	Sodium reduction among specific sub-groups of the population	TS and CS (undivided)	**Declarative data:** self-administered questionnaire targeting 4 food choice behaviours, including a frequency scale for salting habits during meals	In all the countries, women were 6% more likely to report not adding salt; women reported limiting salt to a greater extent than men in 13 countries.<br><br>In France, 60.8% of men and 67.7% of women seek to limit their salt intake.
**Knowledge, attitudes, and beliefs on nutritional norms**							

	(78)<br>Ahn et al., 2017	KOR<br><br>*n* = 3892<br><br>A random sample of Korean participants aged at least 18 years recruited through phone calls, emails, local newspaper advertisements (94.8% women and approximately 45% were college graduates or had higher levels of education)	To investigate the differences in cognitive and behavioural characteristics by different stages of change for reducing sodium intake among Korean participants<br><br>→ **Knowledge, attitudes, behaviours**	Barriers to salt reduction among the general population	FS, CS, TS	**Declarative data:** a self-administered questionnaire with three sections for collecting demographic information, classifying the stage of behavioural change, and obtaining cognitive and behavioural factors related to a reduction of sodium intake.	Concerning the stage of behavioural change, 29.5% of participants were in the maintenance stage and 19.5% in the action stage. Participants in the pre-action stage were 23.3%, 24.0% and 3.7% were in the precontemplation stage, contemplation stage and preparation stage respectively.<br><br>The results suggest that a tailored intervention for reducing sodium intake could be based on the characteristics of each stage (e.g., outcome expectancy; barriers; knowledge; low salt products consumption)
	(55)<br>Arcand et al., 2005	CAN<br><br>*n* = 50<br><br>A random sample of volunteers recruited from an ambulatory heart function clinic	To examine the effect of educational content provided by a dietician on the adherence with a sodium-restricted diet for patients with heart failure; to compare these results with patients group that received nutrition advice through the self-help literature<br><br>→ **Intake, attitude, behaviours**	Awareness as a sodium reduction strategy	Supposedly global but undetermined	**Declarative data:** 3-day food record	Adherence to a low-sodium diet was better initiated and maintained over the long term with considerable support from a dietician. Adherence and maintenance were observed over three months with significant results.

	(53) Brockman, 2019	GBR, USA<br><br>*n* = n/a<br><br>Representative sample studies conducted by Mintel group (private market research)	To evaluate consumer's awareness, knowledge, beliefs, and market strategies regarding salt consumption<br><br>→ **Intake, beliefs, behaviours**	Awareness, knowledge, and low-salt food products reformulation as strategies for salt reduction among the general population	SF, TS, CS	**Declarative data:** Self-administered online survey	Diet-appropriate salt moderation is more prevalent (66%) for participants over 65-year-olds in the UK. In the USA, Black adults more frequently identified salt as a nutrient to be controlled than Hispanic or white people.<br><br>In the USA, respondents indicated that they tried reducing their sodium intake by reducing the salt used in cooking (72%) and seasoning at the table (64%). Only one in four (39%) reported decreasing their consumption of ready-made meals and eating out.<br><br>In England, attention to the nutritional content at the time of purchase is particularly important for the sauce category (50%). This finding is particularly relevant as table sauces and pasta sauces have not followed the trend of reducing their sodium content.<br><br>Women are more attentive to the amount of salt in products than men.<br><br>The increase of sea salt products relied on a more positive perception of such salt as ‘natural’ and healthier than regular salt. This is driven partially by following the trends among gourmet and celebrity chefs. According to the *American Heart Association* (2011), 61% of respondents to a questionnaire considered sea salt as a lower sodium alternative to table salt.

	(73)<br>Cheong et al., 2021	MYS<br><br>*n* = 1047; 798<br><br>A nationally representative sample of Malaysians; the data from this study were obtained from the Malaysian Community Salt Survey (MyCoSS). 1047 respondents completed the questionnaire, and 798 of them provided valid urine<br><br>samples	To evaluate respondents' knowledge, perception, and practice related to sodium intake<br><br>→ **Awareness, beliefs, attitude**	Awareness, knowledge, and self-perceived salt intake as strategies for salt reduction among the general population	Global	**Declarative data:** a questionnaire was adapted from the WHO protocol for population-level sodium determination. Awareness of the impact of excessive salt consumption on health, self-perceived salt intake, and attitude toward reduction were the 3 main themes of the questionnaire.<br><br>**Objective measures:** 24-h urinary sodium excretion test (UNa-24h)	A large part of the respondents knew that excessive sodium intake could cause health issues (86.2%), and more than half of them (61.8%) perceived that they consume just the right amount of sodium. Globally, the logistic regression analysis revealed that excessive dietary sodium intake was not significantly associated with knowledge, perception, and practice related to sodium consumption among respondents. No results are presented for the analysis of the 24-h urinary sodium excretion test.

	(75)<br>Cuadrado-Soto et al., 2019	ESP<br><br>*n* = 239<br><br>A random sample of parents-children dyads from 11 primary schools of 6 different Spanish provinces	To assess the link between parent's salt behaviour and the amount of sodium excreted by their children<br><br>→ **Intake, caregiving practices**	Parental determinants of children's preferences for salty food and DS use	Global	**Declarative data:** self-reported questionnaire for parents about their behaviours related to salt<br><br>**Objective measures:** 24-h urinary sodium excretion test (UNa-24h) for their children	Levels of sodium intake have been associated with parents' salt-related behaviours: the presence of a salt shaker on the table; the use of table salt by the mother<br><br>Children whose families used iodized salt had higher levels than when regular salt was consumed. Iodized salt could have been considered less dangerous or healthier as iodine is known to have health benefits.
	(74)<br>Gray et al., 2014	AUS<br><br>*n* = 151<br><br>A random sample of people with diabetes was recruited from multiple medical clinics; 43% were women, and 82.1% had type 2 diabetes; 43% of the group were retired	To evaluate attitudes, knowledge, and beliefs of health risks of sodium intake among people with diabetes<br><br>→ **Awareness, knowledge, and behaviours**	Sodium reduction among people with risk factors	SF, TS, CS	**Declarative data:** self-administered online survey<br><br>**Objective measures:** 24-h urinary sodium excretion collection and other medical exams: height and weight measurements, blood tests, blood pressure measurement	Only 6% of the surveyed population knew the optimal daily dietary salt recommendations; 23.9% did not feel concerned about reducing their salt intake.<br><br>Most participants were aware that a diet high in salt could contribute to high blood pressure (88.1%) and stroke (78.1%).<br><br>Reading labels is a practice reported by 60 and 80% of each group (type 1 and type 2). However, urine collection from this sample of aware consumers shows that their sodium excretions remain high.<br><br>Eighty-four participants reported using discretionary salt, of which 65 used a salt shaker at the table often (9.3%) and sometimes (33.8%).

	(63)<br>Grimes, Riddell and Newson, 2009	AUS<br><br>*n* = 474<br><br>A random sample of shoppers recruited in two Metropolitan Melbourne shopping centres; 65% women and 64% university education level	To evaluate awareness, knowledge, and attitudes to salt intake and labelled salt information<br><br>→ **Awareness, knowledge, attitude**	Awareness, knowledge, and labelling as strategies for salt reduction among the general population	SF	**Declarative data:** intercept survey containing 27 questions to investigate shoppers' awareness and attitudes to salt consumption	Eighty-eight percent of participants were aware that a diet high in salt could contribute to high blood pressure.<br><br>Sixty-five percent were not able to correctly identify the relationship between salt and sodium. For 40%, sodium and salt were the same substances. Even though 70% of the participants indicated reading the salt content of food products when shopping, this did not always allow people to make a correct choice between two products as the way of quantification often varied on packaging (per serve, per 100 g, reference intakes on food labels).<br><br>In general, snacks (24%), stocks and seasonings (16%), tomato sauces (15%) and condiments (7%) were products (among others) for which consumers were most oriented towards low-salt alternatives.<br><br>Age appeared to be an explanatory variable for regular consultation of the nutritional label at the time of purchase. People aged 45 to 54 81% of times regularly read this information; for the 55-64 age group, the frequency was 80%, while only 50% and 60% were interested in these mentions in the 18-24 and 25-34 age groups. Gender was also mentioned as a differentiating variable. Women declared that they look at the nutritional information on salt more than men (74% versus 58%) and use it to decide on their purchase (75% versus 60%). There was no association between the education level and any of the salt-related shopping behaviours.

	(76)<br>Hoeft et al., 2015	USA<br><br>n = 61<br><br>A random sample of rural Latino caregivers-child-under-their-care dyads with 87% female (41 years old on average); recruited from preschools, community organisations, and local events in Mendota, California	To examine knowledge, beliefs and behaviours concerning caregivers' children's salt consumption, as well as related health implications<br><br>→ **Intake, caregiving practices**	Parental determinants of children's preferences for salty food and DS use	SF, TS, CS	**Qualitative data:** 30 non-directive interviews and 5 focus groups	Three main capacities that children must have in order to add salt in their food at the table on their own have been identified by researchers: (1) the ability to recognize when food tasted too plain or too salty, (2) to have enough dexterity to be able to add a small amount of salt independently, and (3) to understand that excess salt has a bad effect on health.<br><br>Caregivers believe that these skills would be fully acquired by the time they reached 10-14 years of age.
	(111)<br>Huang and Zeng, 2021	CHN<br><br>n = 1610<br><br>Participants aged over 15 years old; recruited to complete an online survey from a Chinese marketing consumer panel; 48.14% being male	To measure participants' engagement with Chinese salt consumption recommendations; to conduct various logistic regressions to determine associations between knowledge, taste preferences, awareness and declared attitudes<br>**Attitude**	Compliance with nutritional guidelines	SF, TS, CS	**Declarative data:** survey containing 57 scaled-response questions or binary questions about socio-demographic, salt knowledge, awareness, attitudes towards low-salt diet, taste preference for salty food	Adoption of labelled salt information, use of salt-restriction spoons, reduced condiment use in home cooking and reduced pickled food intake were far from adopted as only 26.40%, 22.98%, 33.54% and 37.20% respectively, reported following this nutritional guidelines. Respondents who had a higher level of salt knowledge, perceived benefits of salt reduction and participated in nutrition education or training programs on sodium reduction were more likely to read the sodium content of food labels. Adoption of the other measures was largely explained by participants' awareness of hypertension risks and taste preferences.

	(26) Johnson et al., 2017	IND<br><br>*n* = 1395<br><br>An age-and sex-stratified random sample with the mean age of 40 years and with 47% of women; the participants were from urban and rural areas of North and South India	To explore the association between population knowledge, behaviours, and actual salt consumption (overall, and in comparison, with education level)<br><br>→ **Intake, knowledge, behaviours**	Awareness as a sodium reduction strategy. Socio-economic determinants of salt-related dietary changes	Global	**Objective measures:** a single 24-h urine collection and physical measurements to calculate BMI<br><br>**Declarative data:** a questionnaire of 15 questions precising demographics, dietary habits (4 questions), lifestyle behaviours, disease history, medication use, and knowledge (2), attitudes, and behaviours (9) related to salt (adapted from the WHO/PAHO protocol for population sodium determination)	The mean of a24-h urinary salt excretion was 9.27 (8.87–9.69) g/day. Participants reported favourable knowledge and behaviours to minimise risks related to salt, among which were less use of salt while cooking, avoidance of snacks, namkeens, and avoidance of pickles (all $p < 0.003$). The average salt intake was comparable for more educated respondents (9.21, 8.55–9.87 g/day) versus less-educated (9.34, 8.57–10.12 g/day) individuals ($p = 0.82$). There was no significant difference in knowledge and behaviours between more- versus less-educated groups and no clear evidence that the level of education influences salt intake. Several consumer behaviours related to the use of salt during food preparation and consumption of salty products were related to actual salt consumption.
	(77) Kenten, Boulay and Rowe, 2013	GBR<br><br>*n* = 72<br><br>A random sample of people (64 are women) in 6 balanced age groups (from 15 to 84); all recruited via a volunteer database held by the Institute of Food Research.	To examine knowledge and beliefs about salt consumption; to assess differences due to the socio-cultural characteristics.<br><br>→ **Knowledge, beliefs**	Awareness as a sodium reduction strategy	SF, TS, CS	**Qualitative data:** 10 focus groups conducted in London (*n* = 5) and Norwich	Several findings suggest that consumers lack knowledge about (1) the current UK recommended maximum daily intake (6 g), (2) the difference between sodium and salt and how to calculate one another, (3)

	(50)<br>Marakis et al., 2021	DEU; GRC; LKA; POL; SVN; TWN<br><br>*n* = 2459 university students; 42.7 males; median age 21 years	To evaluate students' knowledge, attitude, and beliefs regarding salt and iodine<br><br>**→ Knowledge, attitude, beliefs**	Awareness as sodium reduction strategy and iodine level adequacy	SF, TS, CS	**Declarative data:** multicentre cross-sectional study conducted with the use of a self-administered questionnaire with demographic characteristics and knowledge questions related to salt and iodine.	Several knowledge gaps were identified among European and Asian university students using this questionnaire. Nearly a quarter of the respondents indicated that the recommendations for salt consumption were confusing or contradictory. 80% of the sample were able to say that excessive salt consumption could lead to high blood pressure issues. The questionnaire reveals that cooking salt is much more common than the use of table salt. Discretionary salt is much more common than salty sauces and broths, but one-third of all participants (34%) were not aware of the kind of salt they used.

	(112)<br>Mendoza et al., 2014	CAN<br><br>*n* = 869<br><br>A random sample of members of the Ontario Consumer food panel; 67.3% women	To measure participants' engagement with Canadian salt consumption recommendations; to compare results to age, education level, sex, marital status, and cooking habits<br><br>→ **Attitude**	Compliance with nutritional guidelines	SF, TS, CS	**Declarative data:** self-administered survey measuring the level of engagement in following 23 recommendations to reduce dietary as issued by Health Canada. A Likert scale was used for each recommendation	People over 50 years old found it problematic to avoid salt at the table, rinse canned food, and avoid consuming convenience foods. People under 50 years of age found it difficult to avoid using condiments.<br><br>Respondents with the lowest level of education (under high school) considered cooking with fresh products and avoiding convenience food a challenge.<br><br>Singles report greater disagreement with cooking fresh produce; married people would find it more challenging to use spices as an alternative to salt when preparing meals.<br><br>Men showed an overall lower level of engagement in following health recommendation<br><br>Comparing those who cook or not (50% or more food prepared from scratch), those who cook have a lower level of commitment to not using cooking salt and table salt. The other category would find it difficult not to consume ready-made food.

	(14) Menyanu et al., 2017	GHA, ZAF<br><br>*n* = 10,522<br><br>A national representative sample of adults who took part in the multinational prospective cohort study WHO SAGE	To evaluate awareness, knowledge, and salt use behaviours of Ghanaians and South Africans<br><br>→ **Awareness, knowledge, behaviours**	Compliance with nutritional guidelines	SF, TS, CS	**Declarative data:** this study utilizes two datasets collected during the SAGE-Wave 2 of the WHO SAGE cohort study. Awareness about high salt diet health consequences, personal assessment of the amount of salt consumed, behaviours towards salt were measured	One-third of all respondents were not aware that excessive salt consumption could be dangerous to health, regardless of age or gender.<br><br>Three-quarters (74.9%) perceived that they consume the right amount of salt.<br><br>In the total survey population, 18% reported frequently (always and often combined) adding salt at the table, while the vast majority (91%) reported frequently adding salt when cooking.<br><br>Generational and cultural differences in discretionary salting could be observed as younger adults, and South Africans used table salt more frequently than the older and Ghanaians.

	(79)<br>Mørk, Lähteenmäki and Grunert, 2019	DNK<br><br>*n* = 1030<br><br>Representative sample (regarding sex and age) of Danish	To identify drivers of intention to reduce salt intake and willingness to purchase salt-reduced food products<br><br>→ **Attitude**	Social determinants of salt-related dietary changes	SF, TS, CS	**Declarative data:** self-administered online survey measuring intention to change dietary salt intake, willingness to purchase salt-reduced food products, people's interest in healthy eating, attitudes to the social norms, respondent's awareness of the consequences of salt intake, and discretionary salt use measurement	Personal norms, social pressure, awareness, and general interest in health issues lead to stronger intentions to reduce salt and willingness to buy salt-reduced products. However, a non-significant relationship between the intention to reduce and the purchase of salt-reduced products was observed. Similarly, the willingness to buy salt-reduced products can be observed among people who did not have the intention to reduce their salt intake. Authors suggest that this result can be explained because these is two different types of goals. Salt reduction is an abstract goal, which is difficult to assess and undertake, while the purchase of salt-reduced products is a concrete goal and with concrete rewards.<br><br>Utilitarian and hedonic products category did not imply differences in the purchase of less salty alternatives.

	(51)<br>Newson et al., 2013	AUT, BRA, CHN, DEU, HUN, IND, USA, ZAF<br><br>*n* = 6987<br><br>Population representative samples	To identify barriers to reducing salt through a cross-country comparison study<br><br>→ **Knowledge, beliefs, attitude**	Barriers to salt reduction among the general population	SF, TS, CS	**Declarative data:** self-administered online questionnaire evaluating socio-demographics; actual and perceived levels and sources of salt intake; change in regard to salt reduction; salt usage, knowledge, beliefs and attitude, responsibility for salt reduction, and preferred methods of communication about salt and salt reduction	The average salt consumption per day was 9.5 g. The vast majority of participants rated their consumption as satisfactory, while Germans, Austrians, and Chinese perceived too high salt intake.<br><br>Meals eaten at home were the major contributors to the total intake, accounting for 83%, with 51% from manufactured products, 25% added at cooking (more for India), and 7% of salt added at the mealtime. Cooking salt was considered to be the most essential contributor to sodium intake, according to the respondents.<br><br>Concerning knowledge, 55% of the survey population indicated that they did not know the recommended sodium intake, and 32% reported incorrect values: 34% of Chinese respondents knew the guidelines, compared to only 3% in the USA.<br><br>Overall, 22% of participants said they usually or always add salt before tasting.<br><br>Regarding beliefs (7-point rating scale), salt was believed a contributor to iodine intake (4.4) and a replacement of what is lost in sweat (4.2). In China, salt was considered necessary for physical activity (4.7) and stimulating the appetite (4.3).<br><br>Participants generally indicated that they were interested in knowing which foods are the most important sources of salt in a diet (59%) and how to reduce the use of cooking salt (52%). To disseminate these messages, television (41%), health professionals (35%), and food packaging

							(22%) were the most popular media to do so.

	(13)<br>Pesantes et al., 2017	PER<br><br>*n* = 98<br><br>14 focus groups (six villages with a predominantly urban population in Tumbes, Northern Peru) divided along with gender and age groups: women 20-44, women 45-65, men 20-44 (substituted by 7 interviews due to nonattendance), men 45-65. Five interviews with health workers from the villages with a primary healthcare facility and 20 interviews with people with hypertension	To scan local explanations about the relationship between salt consumption, diet, hypertension, and dietary changes to reduce salt intake.<br><br>→ **Attitudes, opinions, practices**	Sodium reduction among the general population	SF, TS, CS	**Qualitative data: s**emi-structured interviews and focus groups about perceptions of hypertension, diet, salt consumption, and health. Three different interview guides (for focus groups, health workers, and people with hypertension) tackled the knowledge about hypertension, views on salt consumption, and expectations of a new product. For individuals with hypertension, questions on causes and management of hypertension and dietary changes were added. For health workers, questions about local health needs and beliefs were added. Interviews and focus groups were transcribed and analysed using software Atlas-ti 7.1.	The study was used as a formative phase for an intervention oriented to promote salt substitutes containing less sodium than regular salt. There was confusion on how high-sodium diet influences health status; hypertension was associated with emotional troubles, changing diet was perceived as complex and negative. Key insights: information about the links between salt use and hypertension is poor; audience segmentation would not influence social marketing campaign; local understandings of disease should be taken into account
	(66)<br>Rhodes et al., 2016	AUS<br><br>*n* = 114<br><br>A random sample of participants from 27 three-generation multicultural families living in Australia	To examine the intergenerational transmission of eating behaviour between generations of multicultural families<br><br>→ **Behaviours**	Family as an instance of socialisation for healthier eating habits	SF	**Qualitative data:** semi-structured family interviews	Regardless of ethnic background, women in each generation influenced fruit and vegetable intake and controlled food purchases, negotiating the consumption of snacks and using food as a prerequisite for conditional treats. Grandparents and children shared relationships that skipped the parents' generation, influencing each other's behaviours by being reciprocal gatekeepers

	(57)<br>Saje et al., 2020	ETH<br><br>*n* = 286<br><br>A random sample of participants living in Addis Ababa, the capital city of Ethiopia; recruitment method is unclear	To estimate the consumption of dietary sodium and potassium; to assess salt-related knowledge, attitude, and behaviour among adults in Addis, Ababa, Ethiopia<br>→ **Knowledge, attitudes, behaviour**	Knowledge, attitude, and behaviours as a sodium reduction strategy		**Objective measures:** 24-hour urine collection tests.<br><br>**Declarative data:** Twenty-four-hour diet recall and questionnaire. Knowledge questions included the daily maximum recommended intake of salt, sources of salt, and the relation between excessive salt intake and health problems. Questions to assess attitude included the self-perceived consumption of salt and attitude toward the importance of lowering dietary sodium consumption. Salt-related behaviour was evaluated using questions such as the behaviour of adding salt and engagement to salt reduction	Only five (1.8%) of participants knew that there is a recommended maximum amount for daily salt consumption.56.3% of the respondents perceived that their salt intake is just the right amount, and 58.1% responded positively to the importance of lowering salt intake. More than 75% of the participants do not add salt at the table, and 90.1% are aware of the effect of high salt intake on health.

	(58) Silva-Santos et al., 2022	PRT<br><br>*n* = 97<br><br>Public university employees without health issues, recruited via routine occupational health consultations at the University of Porto	To verify whether an intervention to reduce added salt during cooking changed knowledge, attitudes, and behaviour towards salt. To analyse changes in the main sources of salt	Knowledge, attitude, and behaviours as a sodium reduction strategy	Global	**Objective measures:** 24-hour urine collection tests.<br><br>**Declarative data:** Twenty-four-hour diet recall to estimate energy intake, sodium intake, and major sources of sodium, including added salt. And a questionnaire to assess behaviours, knowledge, and attitudes related to added salt.	In the end of the intervention, there was an increase in participants who stopped eating processed foods and adding table salt; who used spices in addition to salt and recognized the importance of reducing salt in the diet. (compared to the control group).

	(33) Wentzel-Viljoen et al., 2017	ZAF<br><br>*n* = 550<br><br>A random sample of Black women aged 18-55 years at baseline and 477 in the follow-up survey	To evaluate the impact of a mass-media campaign (Salt Watch media campaign) on knowledge, attitudes, beliefs, and intentions to reduce salt consumption<br><br>→ **Knowledge, attitudes, beliefs**	Awareness as a sodium reduction strategy	SF, TS, CS	**Declarative data:** administered questionnaires at baseline, including questions on the socio-demographic characteristics of the participants, their knowledge (health risks), attitudes and beliefs, behaviour (declaring controlling their salt intake) past and current behaviour to salt intake. The follow-up survey also included a "stage of change" questionnaire to measure participants' current interest in reducing their salt intake.<br><br>The public awareness campaign consisted of television and radio advertisements where a well-known South African medical doctor and media personas emphasised the message that South Africans are consuming too much salt. Explaining the health risks of such consumption and expressing the need to reduce discretionary salt. The mass-media campaign also included various supporting activities (talks, brochures, emails, editorials).	In the follow-up survey, 77.8% of the participants reported that they had seen a Salt Watch campaign.<br><br>Post-intervention, more participants reported that they were taking steps to control their salt intake, and the indicators of knowledge, attitudes, and behaviour changed. Reported salt practices after the campaign indicated significant behaviour changes regarding the use of cooking salt (45.2% at baseline versus 59.1% during follow-up survey reported not adding salt when cooking) and table salt (14.2% vs 20.1%), with a higher use of herbs and spices used as DS alternative (70.0% vs 77.8%), compared to other salt reduction behaviours, such as buying low salt alternatives, looking at the salt or sodium labels on food, avoiding eating out, etc.
**Salt practices associated with dietary or medical regimes**							

	(83)<br>Adriouch et al., 2017	FRA<br><br>*n* = 26,750<br><br>In two cohorts: 50% diagnosed with a cardiometabolic disease and 50% controls matched by sex and age; 35–70-year-olds	To evaluate and compare cardiometabolic disease patients and their health-matched controls in terms of compliance to the dietary salt intake recommendations<br><br>→ **Intake, attitude**	Sodium reduction among people with risk factors	Global	**Declarative data:** 3 web-based 24-h records	The recommendations on salt showed the lowest compliance among both groups, with better compliance from the control group than from the patients.<br><br>The average salt intake was 8.8 g/day for a control group and 9.2 g/day for hypertensive patients.<br><br>As for the actors responsible for reducing daily salt intake, participants indicated that they were themselves responsible (5.9 out of 7 scores), followed by responsibility of food manufacturers (5.0), restaurants (4.7), family, and friends (4.1). The government and international organisations came last in this ranking.
	(85)<br>Bournez et al., 2019	FRA<br><br>*n* = 18,258<br><br>A representative sample of children (aged 3 to 10 months) and their mothers; recruited in a random sample of 349 maternity units in France	To compare the frequency of use of added sugar, salt, and fat during the complementary feeding period following infant caregiving and cultural, culinary practices<br><br>→ **Intake, caregiving practices**	Compliance with infant feeding nutritional guidelines	TS and CS	**Declarative data:** self-reported questionnaire conducted as part of the French ELFE study. DS use was recorded on a five-point scale from "never introduced" to "always or almost always introduced."	Early complementary feeding introduction (before four months) was positively related to the use of added salt.<br><br>Baby food was used without any modification regarding salt.<br><br>Children who ate little at two months were more likely to get added salt in their food if their mother were in an "insisting or trying later" compared to "non-insisting" caregiving.

	(54)<br>Chung et al., 2006	AUS, USA<br><br>*n* = 68<br><br>A random sample of participants was recruited from 3 medical clinics (41 men and 27 women). American subjects were similar to the Australians in socio-demographics and clinical characteristics (no intention of comparative cross-country analysis)	To examine gender-related differences in adherence to a sodium-restricted diet among patients with heart failure<br><br>→ **Awareness, attitude**	Sodium reduction among people with risk factors	Global	**Declarative data:** adherence to the diet, knowledge, and barriers, as well as sociodemographic and clinical characteristics, were obtained through a questionnaire. The participants also provided a food diary<br><br>**Objective measures:** 24-h urinary sodium excretion collection	Women were the most adherent to SRD with regard to the level of sodium in their urine 24 hours later; they were better at recognizing the effects of excess salt on their health problems.<br><br>There was no gender difference in barriers to adopting an SRD; only 49% of patients understood and knew how to follow it. Seventy-five percent had difficulty adhering to their diet when eating out, 69% felt that salt-reduced food did not taste good, and 72% indicated that their favourite foods were not low in salt.<br><br>Restaurants, friends and family (54%), the cost of salt-reduced products (47%), and the time needed to prepare less salty meals (38%) appeared as barriers.
	(86)<br>Dyett et al., 2013	USA<br><br>*n* = 100<br><br>A random sample of respondents (aged 25-75) who have practised vegan lifestyle for at least 9 months; recruited via printed and electronic advertising and living in the different U.S. States	To examine the effects of respondents' daily vegan diet on their nutrient intakes<br><br>→ **Intake, DS use**	Potential dietary deficiencies related to a restrictive diet	Global	**Declarative data:** self-administered survey and telephone interviews using 3-day diet recalls.	3% of respondents reported a high salt use, 35% adopted this diet for health-related beliefs.

	(84)<br>Henson et al., 2010	CAN<br><br>*n* = 446<br><br>A random sample of people recruited in a mall in Guelph, Ontario; 50.4% of women	To identify the difficulties of complying with nutritional recommendations for the prevention of heart problems<br><br>→ **Attitude**	Compliance with nutritional guidelines	SF, TS, CS	**Declarative data:** Intercept survey covering twelve dietary guideline items, including two on salted products and about discretionary salt use. Participants were asked to score each item according to the extent to which their diet had complied over the last year (seven-point scale from no at all to completely).	The least difficult dietary recommendations to comply with, according to the participants, were the consumption of poly- and unsaturated oils, the control of discretionary salt, the avoidance of products containing trans fats, and the use of specific cooking methods.<br><br>The group of men aged 35 years and over responded that it was relatively easy for them to avoid foods containing high salt levels compared to the rest of the sample.<br><br>The results suggest that dietary changes involving major significant food habits transformations are considered more difficult to undertake (i.e., avoiding red meat) than recommendations that offer alternatives such as choosing a different cooking fat, a different cooking technique, etc.

	(113)<br>Li et al., 2022	USA<br><br>*n* = 15,352<br><br>Adults with hypertension who took part in the multinational prospective cohort study NHANES	To examine the effect of refusal to add salt or the usage of different salt types on hypertension<br><br>→ **Intake, DS use**	Potential health hazards due to not using salt or using different salt types at the table and when cooking	TS, CS	**Declarative data :** 24-h dietary recall with self-reported DS use at the table and during cooking. Medical conditions data from NHANES study program	Patients with other salt intake (lite salt or salt substitute) were associated with a higher risk of hypertension or hypertension and stroke. People using substitutes may use more of them, resulting in a high sodium intake.<br><br>Not adding salt products at the table and excessive use of salt low on sodium might cause salt-sensitivity and hypertension.

	(81) Lofthouse, Te Morenga and McLean, 2016	NZL<br><br>*n* = 11<br><br>A random sample of participants, including 7 women; recruited through word of mouth, social networks, and flyers placed at the University of Otago, Dunedin; with an average age of 39.2 (± 12.8); 8 participants reported they had a postgraduate university degree	To examine the feasibility of adherence to a low-sodium diet among healthy adults<br><br>→ **Attitude, behaviours**	Sodium reduction among the general population	SF, TS, CS	**Objective measures:** first, intake data were collected using a questionnaire, and a 24-h urine collection was conducted. Participants were then involved in a four-week nutrition intervention to help them achieve a low sodium diet while getting weekly clinics advice and support.<br><br>**Qualitative data:** a semi-structured interview was then conducted to gain insight into participants' personal experiences and perceived barriers or facilitators to adhering to the diet.	The clinical experiment has led to an 18% reduction in excreted sodium after 24 hours (53% after three days on average).<br><br>The participants were forced to cook more at home, which involved making a menu list in advance. Unfortunately, this transition to more home cooking did not happen for time-pressed participants.<br><br>Five participants indicated that they counted their salt intake and tried to distribute it over the day.<br><br>Five participants used an alternative to salt, a product formulated with a high proportion of potassium chloride and less sodium chloride. These people reported using it in smaller amounts than they would have with conventional salt, only at the table and not while cooking.<br><br>All participants mentioned the difficulties of eating meals outside the home.<br><br>Social support may have played an essential role in overcoming difficulties in adopting the low-salt diet. Some participants mentioned the trade-off between cooking without salt and letting other household members add salt at mealtimes.<br><br>Several people found it difficult to understand nutritional information on the products.

	(80)<br>Pillay et al., 2017	FIJ<br><br>*n* = 169; 272<br><br>A representative sample of the adult population in Fiji (contacted via telephone and through home visits) in two cohorts: 169 participants at baseline and 272 at 20 months (28.2% and 22.4% participation rates)<br><br>Mean age: 42%<br>Even distribution of sex and ethnicity.<br>40% with the education of o primary and secondary levels.	To evaluate the impact of an intervention to reduce daily salt intake on salt intake (measured through 24-h urine samples) and to ensure that this program does not affect iodine intakes simultaneously.<br><br>→ **Intake, attitude**	Assessment of a salt reduction intervention	Global	**Objective measures:** 24-h urinary sodium excretion collection and physical measurements. At 20 months, 131 urine collections of non-pregnant females and child-bearing age were also collected.<br><br>**Declarative data:** a survey administered by trained Public Health nurses and trained research assistants for sociodemographic data collection (age, sex, education level, ethnicity)<br><br>The data were obtained at the baseline and after the start of the intervention at 20 months.<br><br>The intervention consisted of the population-wide and multi-sectoral<br><br>program targeting (i) food manufacturers and retailers (e.g., products reformulation), (ii) the main Fiji hospitals (e.g., educating food service staff in the preparation of low salt meals) (iii), and consumers through health workers, media and community leaders.	The average salt intake was 11.7 g/day at the baseline and 10.3 g/day after 20 months. No significant reduction in salt intake has been observed.
**Salt materialities: interactions, and contexts**							

	(114)<br>Ayya and Beauchamp, 1992	USA<br><br>*n* = 10<br><br>Volunteers aged 19-32 years	To test how optimal salt levels in food are associated with the use of salt during the meal<br><br>→ **Preference**	Personal acceptability of salt reduction	CAS	**Collected data using experiments:** vegetable broth prepared with seven different concentrations of salt under controlled conditions. Subjects' preferred salt levels were determined before, and after two meals, one of which was high on salt and the other was low.	Preferred salt levels in soup declined significantly following the high-salt meal. No change was observed during the low-salt meal.
	(115)<br>Bertino, Beauchamp and Engelman, 1986	USA<br><br>*n* = 19<br><br>A random sample of participants (aged 17 to 25), including 6 females; recruited from the University of Pennsylvania through an advertisement in a student newspaper	To examine the effect of salty taste exposure on salt preferences<br><br>→ **Preference**	Personal acceptability of salt reduction	CAS	**Collected data using experiments:** vegetable soup prepared with nine different concentrations of salt under controlled conditions. Each soup was served 3 times, and the subjects were instructed to assign numbers in direct proportion to the intensity of saltiness and pleasantness in comparison to the preceding stimulus	Salt preferences can be modified by dietary salt manipulation. However, the increase of salt intake produces a higher salt preference more quickly than the decrease for the liking of salt-reduced products.
	(31)<br>Blanco-Metzler et al., 2021	CRI<br><br>*n* = 91<br><br>Adults: 11 cultural managers, 80 key informants (26 participated in interviews and 54 in 12 workshops)	To study the food practices and perceptions related to excessive consumption of salt when cooking and outside the home in a study population sample that considers the intergenerational and sociocultural diversity of Costa Rica.	Social and cultural determinants of food practices and perceptions of household cooking and eating out salt consumption	SF, TS, CS	**Qualitative data:** a semi-structured interview; investigative workshop with cultural experts and key informants from the communities; demonstrations carried out with participants who agreed to prepare dishes that they usually eat at home as a family; participant observation	Age factors were suggested as being the main differentiators, in the use of salt, seasonings, and condiments, beyond the cultural and geographical differences in Costa Rica.<br><br>Most individuals could not calculate the salt added during food preparation and could not estimate hidden salt in a variety of foods. Salt is an essential component of foods with strong cultural roots. It is associated with emotions and the caring of the family.

	(94) Bobowski, Rendahl and Vickers, 2014	USA<br><br>*n* = 83<br><br>A random sample of subjects recruited through a database of students and staff of the University of Minnesota	To evaluate the effectiveness of a step-by-step salt reduction process versus an abrupt reduction in relation to consumer acceptability of a low sodium food<br><br>→ **Preference**	Personal acceptability of salt reduction	CAS	**Collected data using experiments:** after an initial tasting test, subjects were divided into two groups based on their motivation to reduce dietary salt intake and their hedonic sensitivity to salt. Experimental protocol included tomato juice served during a 16-weeks trial, abruptly or gradually decreasing in salt from 640 mg sodium per 237 ml to 136 mg	There was no difference in preference with regard to reduction strategies. However, an abrupt reduction leads to an equally abrupt decrease in the appreciation of tomato juice.<br><br>People who liked the least salty soup at the first tasting test did not experience any discomfort in both gradual and abrupt decrease situations.
	(59) Brembeck and Fuentes, 2017	SWE<br><br>*n* = 19<br><br>A random sample of primiparous mothers (aged 20-25); recruited from a local health clinic of Falköping, a middle-class-dominated urban area (in terms of income, education, and age of mothers).	To examine the effect of integration of convenience baby foods on the increase of salt use<br><br>→ **Intake, caregiving practices**	Strategies of healthy nutritional weaning process	TS and CS	**Qualitative data:** non-directive interviews	Different strategies have been adopted during the weaning period to teach babies to appreciate the family's food while respecting their needs of a low-sodium diet: TS replaces CS; CS is added after a portion of food has been set aside for the baby. In addition, parents would accept to eat tasteless food for the sake of convenience and so that they can eat together with the same meal.

	(28)<br>De Kock et al., 2016	ZAF<br><br>*n* = 432<br><br>A random sample of urban Black South African consumers in the Tshwane metropolitan area; 51% female; the average age of 35.2 years (±12.3 years)	To evaluate liking, salt taste perception, and use of table salt when consuming reduced-salt food<br><br>→ **Preference, behaviours**	Personal acceptability of salt reduction. (Behavioural response)	CAS and TS	**Collected data using experiments:** chicken stews prepared under controlled conditions with 4 differently salted stock cube powders. Participants were randomly allocated to consume one of the four chicken stews. Liking, salt taste perception, and the use of table salt were measured in order to observe how consumers compensate for controlled salt reduction by adding salt back by themselves. To assess the consumption of added table salt at the time of the meal, the salt shakers were previously weighed on a digital scale	Nineteen percent of consumers who tasted the low-salt chicken stew fully compensated by adding table salt, reaching initial salt concentration of regular commercial stock cube powders. Generally, as the meals contained reduced sodium stock cubes, participants increased the dose of table salt.

	(49)<br>Dötsch-Klerk et al., 2015	GBR, USA, NLD<br>*n* = 1724 (UK NDNS survey; adult population 19-64 years old); 7227 (US NHANES survey; total population 2-80 years old); 750 (Dutch DNFCS survey; young adult 18-30 years old)	To assess the potential impact of reformulated foods on the population salt intake<br><br>→ **Intake**	Food products reformulation as strategies for salt reduction among the general population	SF	**Declarative data:** data modelling using survey data from 3 different national food consumption surveys and recalls, typical menus, and composition tables. The average total daily salt intake was compared with two different salt reduction scenarios (6g of salt and further reduction towards 5g of salt per day) to evaluate the impact of reformulation of foods on the population salt intake	The expected impact of salt reduction in food products is different between countries since the contribution of salty food to total daily intake is not the same. In Spain and the US, results suggest that raising awarenessx² of the need to reduce intake of food products that contribute most to their intake would be a better strategy. On the other hand, in China and South Africa, discretionary salt is the major contributor to total daily salt intake and should be targeted.<br><br>High salt users show more significant sodium intake reductions. For example, salt would be reduced by 22 to 29% using the 6g/day product criteria and by 28-33% reduction using the 5g/day product criteria.

	(60)<br>Farleigh, Shepherd and Wharf, 1990	GBR<br><br>*n* = 1859<br><br>The staff of a local company who completed the questionnaire on the first day of the study (no details are provided); the local company canteen was serving between 2200 and 2800 meals daily with a mean of 2426	To examine the effect of manipulation of salt shakers hole size on table salt use<br><br>→ **Behaviours**	The impact of material environment on salt usage	TS	**Collected data using experiments:** experimental protocol took place at the workplace canteen serving approx. 2400 meals, the daily use of salt shakers was measured for 10 weeks. Salt shakers were modified by varying their hole diameter and the area on a cap. All salt shakers were weighed after meals to determine the amount of salt consumed by consumers previously counted by using a questionnaire. The second data set included the shaking time of the salt pot, which was estimated by comparing the total quantity consumed with the flow rate observed in the laboratory	Despite a 10-day exposure to each new salt shaker, participants used more table salt when the salt pots with the largest holes were supplied. However, the use of the salt pots with the largest holes leads to a shorter time of shaking. The authors suggested that table salt use is subject to both sensory and habitual control.

	(94)<br>Ferber and Cabanac, 1987	FRA<br><br>*n* = 10<br><br>A random sample of men adults; average age 22.4 years (±0.67)	To examine the effect of noise during a tasting session on the preference for sweet and salt<br><br>→ **Behaviours**	The impact of material environment on salt usage	CAS	**Collected data using experiments:** this study consisted of two experiments. First, men were exposed to four different noise conditions from silence control to loud music (90 dB), while they tasted five differently salted solutions. Participants had to rate the pleasure/displeasure aroused by the five stimuli. The second experiment involved asking participants to mix solutions in order to obtain the most pleasant saltiness	No change was observed regarding salty solutions when affective rating and preferred concentrations were compared to the control substances.
	(88)<br>Frye and Demolar, 1994	USA<br><br>*n* = 80<br><br>A random sample of 49 female and 31 male college students (17 and 22 years old)	To compare salt preference between men and women; to assess if some stages of the menstrual cycle affect salt preference<br><br>→ **Preference**	Physiological determinants of salt preference	CAS	**Collected data using experiments:** preference rating for more or less salted popcorn under controlled condition	Men liked mildly salty popcorn more than women.<br><br>Women in their menstrual week are more likely to prefer salty foods (popcorns) than those tested during their follicular, luteal, or ovulatory cycle stages.

	(91)<br>Girgis et al., 2003	AUS<br><br>*n* = 110<br><br>A random sample of volunteers among the staff of the Royal North Shore Hospital; 79% female; the average age of 39 years old (range 22-62)	To evaluate the effect of gradual reduction of salt quantity in bread on liking and salt taste perception<br><br>→ **Taste perception, preference**	Personal acceptability of salt reduction	CAS	**Collected data using experiments:** During the six consecutive weeks, the volunteers were offered bread containing the usual proportion of sodium (2 g per 100 g of dough). This period was followed by six weeks, during which the amount of salt was gradually reduced by 5% each week. The study also included a control group (half and half) that did not consume salt-reduced bread during the last 6 weeks of the experiment.	Reducing the amount of sodium by a quarter is possible and does not cause any change in the acceptability compared to the control group.<br><br>This reduction did not affect the scores for flavour.<br><br>Participants did not report perceiving differences between the pieces of bread during the weeks of reduction. Therefore, the liking of bread remained the same.
	(116)<br>Herbert et al., 2014	GBR<br><br>*n* = 15<br><br>A random sample of participants recruited from the student population at the University of Bristol	To evaluate the memorization of the sensory characteristics of recently consumed food containing salt<br><br>→ **Taste perception**	Physiological determinants of salt consumption	CAS	**Collected data using experiments:** remembered salt concentration and hedonic evaluation of twelve salt concentrations in soups prepared under controlled conditions. Four exposure conditions were defined (regular, reduced, variable and declining).	The various previous interactions with test-soups had little effect on taste memory. However, participants who received the non-light soups remembered that they were saltier than those presented during the last tasting session.

	(89)<br>Leshem, 1998	ISR<br><br>*n* = 50<br><br>A random sample of children-mothers dyads recruited by word of mouth; 24 girls aged 11-18.5 years and 26 boys aged 12-19 years	To assess the relationship between the prenatal and infantile mineral fluid loss and salt preference during adolescence<br><br>→ **Preference**	Physiological determinants of salt preference	CAS, SF, TS, CS	**Declarative data:** evaluation of the history of mineral fluid loss was obtained through a questionnaire submitted to the mothers. The frequency of maternal vomiting during pregnancy, vomiting and infant diarrhoea in the first months of life were so measured. Measures of the avidness for salt were obtained via interviews about taste and dietary habits.<br><br>**Collected data using experiments:** evaluation of the preferred concentration of sodium in soup.	Mineral fluid loss during gestation and childhood contributes to a long-term avidness for salt; the greater was the incidence of mineral fluid loss, the greater was the avidness for salt among adolescents.

	(96)<br>Leshem, 2017	ISR<br><br>*n* = 140<br><br>A random sample of students from the University of Haifa aged 24.2 (± 0.6); 78% women	To verify whether or not salt intake increases during the summer period due to mineral fluid loss through sweating<br><br>→ **Preference**	Physiological determinants of salt consumption	CAS	**Collected data using experiments:** participants were presented with two cups of tomato soup (one unsalted and the other one with sodium) and informed that one cup was flavoured. Using the method of mixtures, the participants were asked to add and mix from either cup until the mixture was tasty enough. The two controlled cups were then weighed to determine the salt concentration of the third one.<br><br>**Declarative data:** a questionnaire was also administered to collect dietary intake habits, demographics, smoking habits, health and exercise data. Participants were tested in both winter and summer.	Under the conditions of the study, the results did not support the hypothesis that there are differences in salt intake between winter and summer among the young, healthy people in the sample.

	(90)<br>Leshem, Abutbul and Eilon, 1999	ISR<br><br>*n* = 21; 21<br><br>A random sample of male sport students practising 30-60 minutes of sport two to three afternoons per week for at least 3 months prior to the study date; a further 21 students who did not meet the criteria of sports practitioners were selected as a control group	To measure how exercise increases the preference for salt<br><br>→ **Preference**	Physiological determinants of salt preference	CAS	**Collected data using experiments:** each group of exercisers and controls were presented with two cups of tomato soup (one unsalted and the other one with sodium) and informed that one cup was flavoured. Using the method of mixtures, exercisers were asked to add and mix from either cup until the mixture was tasty enough. The two controlled cups were then weighed to determine the salt concentration of the third one. This protocol was repeated before exercise, 12 hours after, and a final time 24 hours post-exercise. Participants were also encouraged to eat freely commercial salty snack items. The number of morsels eaten was recorded	The preference for a higher salt concentration in tomato soup did not increase in the control group but increased significantly after a sports session in the exercisers group. The change occurred immediately (30 minutes post-exercise) and lasted up to 12 hours after the exercise session.<br><br>The salt content of the soup increased by about 50% between the pre-and post-workout tests. Thus, this study confirmed that attraction to salt increased as its concentration in the body decreased; the suggestion was that sweating might be a mechanism for this phenomenon.

	(117)<br>Lucas et al., 2011	AUS<br><br>For two separate studies<br><br>*n*1 = 56 students enrolled in a science course, including 48 females<br><br>*n*2 = 22 participants (15 female) recruited via emails and advertising placed around the University of Deakin, Melbourne	To assess if the sodium concentration in a dish (hash browns) influences its liking and consumption<br><br>→ **Preference**	Personal acceptability of salt reduction. (Hedonic response)	CAS	**Collected data using experiments:** study 1 was carried out over 2 sessions and aimed to determine detection and recognition thresholds for sodium concentrations contained in hash browns. Study 2 was performed over 2 weeks during 5 sessions. The 1st session was to determine taste thresholds. The 4 remaining sessions consisted of free lunch-time consumption of one of the 4 varieties of hash brown prepared under controlled conditions. The objective of this second study was to measure levels of liking.	In both experiments, the quantity of salt in hash browns did not affect the appreciation of the dishes.

	(64) McMahon, Webster and Brimblecombe, 2017	AUS<br><br>*n* = 25<br><br>Remote Indigenous community stores in the Northern Territory, Western Australia, and Southern Australia are managed by community associations. 11 control stores and 15 intervention stores	To investigate whether 25% sodium reduction in a top-selling bread affected sales in 26 remote Indigenous community stores in Australia. To examine whether this intervention would significantly impact the overall intake of sodium through foods and drinks.<br><br>→ **Consumer practices. Commercial value**	Sodium reduction among specific sub-groups of the population. Consumer acceptance of sodium reduction. The commercial viability of sodium reduction.	SF	**Collected data using experiments:** non-randomized control study of intervention group (reduced salt) vs control group (regular salt) in remote Australian community stores. Following a 23-week baseline period, for 12 weeks, the regular-salt bread (400 mg Na/100 g) was distributed to 11 control stores, and 15 intervention stores received the reduced-salt version (300 mg Na/100 g). Sales data were collected to study differences between groups in change from baseline to follow-up (effect size) in sales (primary outcome) or sodium density, analysed using a mixed model. Analyses were performed using mixed models that included different periods, and groups using STATA version 13.1.	The 25% salt reduction in a top-selling bread did not affect sales in remote Indigenous community stores. If achieved across all loaves of bread, estimated salt intake in remote Indigenous Australian communities would be reduced by approximately 15% of the magnitude needed to achieve population salt targets

	(8)<br>Menyanu et al., 2021	GHA; ZAF<br><br>*n* = 1202<br><br>A national representative sample of adults who took part in the multinational prospective cohort study WHO SAGE	To evaluate the impact of an intervention to reduce daily salt intake on salt intake (measured through 24-h urine samples) and to ensure that this program does not affect iodine intake at the same time.<br><br>→ **Intake**	Assessment of a salt reduction intervention and evaluation of the impact of this intervention on iodine intake	Global	**Declarative data: t**his study utilises two datasets collected during the SAGE-Wave 2 of the WHO SAGE cohort study. Awareness about high salt diet health consequences, personal assessment of the amount of salt consumed, behaviours towards salt were measured<br><br>**Objective measures:** 24-h urinary sodium excretion test	In the sample of South African adults surveyed after the introduction of a salt-reduction policy, those with low salt intake (<5g/d) had inadequate iodine intake. This negative relation was not observed in the sample of Ghanaian adults. On the other hand, South Africans with salt intake above 5 g/d had optimal estimated iodine intake. Ghanaians had optimal intake across all salt intake categories.

	(95) Odom et al., 2017	USA<br><br>*n* = 4170; 4127<br><br>Two national research panel surveys were conducted in 2012 and 2015; 51.2% female; 77.3% of 31 years or older	To examine the difference between the two surveys in agreement with broad-based actions to limit sodium as well as environment-specific policies (e.g., in restaurants, in manufactured foods, in school cafeterias) with the same objective.<br><br>→ **Attitude**	Consumer acceptance of sodium reduction	SF, CS	**Declarative data:** a cross-sectional study measuring consumer agreement with or support for broad-based actions to limit sodium in foods (five-point Likert scale for statements agreement evaluation), demographics and health characteristics (e.g., age, race-ethnicity, household income, education level, BMI), consumer desire to eat less sodium (five-point Likert scale).	No change between the years occurred regarding agreements with broad-based actions in restaurants (45.9% agreed in 2015) and manufactured foods (56.5% agreed in 2015). From 2012 to 2015, there was a significant (^<0.0001) increase in respondents who supported policies to lower sodium in school cafeterias (80.0 to 84.9%), workplace cafeterias (71.2% to 76.6%), and quick-serve restaurants (70.8% to 76.7%).

	(65)<br>Payne Riches et al., 2019	GBR<br><br>*n* = 947<br><br>A random sample of participants was divided into two groups (476 and 471 people) recruited via two channels: a database of volunteers with different health problems and a UK-wide panel from a market research agency; 42% were female	To examine the effect of the offer to change a product for a similar but less salted on final shopping choices<br><br>→ **Behaviours**	Sodium reduction among people with risk factors	SF	**Collected data using experiments:** experimental study using a virtual supermarket where internet users (hypertensive study participants) had to buy 12 food products (including dressing, butter and spreads categories). One group was presented with minimally less salt (MLS) alternatives of 5-20%. The second group was offered alternatives reduced in salt to more than 20% (substantially less salt, SLS) as well as MLS. The groups were compared with the amount of salt contained in their virtual shopping baskets (g/100 g of products) and compared with the amount of salt present in their basket of products initially selected (before the presentation of alternatives). The number of products that were changed for salt-reduced alternatives was also measured.	The offer of less salty alternatives reduced the average amount of salt in the shopping basket of both groups (30% less in the second group).<br><br>Between the initial and final baskets, the amount of fat and sugar also decreased slightly, as did the cost of the basket. The proportion of product swaps was the same for both groups.<br><br>Among those who accepted the most product changes, the authors mention health-concerned food shoppers, people advised by a health professional, or those who often look at the nutrition labels for salt.

	(93) Shepherd, Farleigh and Wharf, 1987	GBR<br><br>*n* = 32<br><br>A random sample of subjects (16 male and 16 female)	To assess preferences for salt in different foods and their relationship to sodium availability<br><br>→ **Taste perception, preference**	Food physico-chemical interactions with salt preference	CAS	**Collected data using experiments:** after several taste tests, participants were asked to indicate their personal ideal NaCl concentration for four different foods: soup, bread, boiled potatoes, and meat pie, all prepared under controlled conditions	The nature of food and how it was prepared had an influence on salt perception.<br><br>Among the four foods, participants preferred the lowest salt concentrations when eating boiled potatoes, followed by eating soup. Salting a potato during cooking was equivalent to penetrating the upper layers of the vegetable with salt, making its perception much stranger than if an equal amount of NaCl were evenly distributed throughout the potato.<br><br>The soup was composed of water, a carrier for salt and hence was available to the taste receptors more easily.<br><br>Finally, bread and meat pie were the foods for which high salt levels were most preferred, presumably due to their solid matter where sodium is evenly distributed

	(61)<br>Shepherd, Farleigh and Wharf, 1989	GBR<br><br>*n* = 266<br><br>A random sample of students and staff at the University of East Anglia responded to a questionnaire asking if they usually added salt to food at the table, tasted their food before salting it, etc.; sixteen of the survey respondents took part in the study (7 female)	To evaluate table salt compensation for reduced salt meals<br><br>→ **Preference, behaviours**	Personal acceptability of salt reduction. (Behavioural response)	CAS and TS	**Collected data using experiments:** to assess the use of table salt, participants ate standard meals in the controlled conditions in the laboratory. Over the 10-days experiment, the dishes offered on 5 days were either not salted (containing 0.46 g of NaCl) or were salted, up to 5.09 g. All participants had access to table salt	The consumers did not fully compensate for the difference in salt removed from the meal. For the salted dishes, volunteers added an average of 0.36 g of salt, compensating for 22% of the difference. The gender of the participants did not affect the results. 69% reported salting their food before tasting. The possible explanations were the expectation of the amount of salt in the dish and the strength of habits.
	(87)<br>Siro et al., 2021	ZAF<br><br>*n* = 250; 312<br><br>Pregnant women from the NuPED study and the STRIPE-SA study conducted in South Africa	To evaluate the impact of an intervention to reduce daily salt intake on iodine intake of pregnant women<br><br>→ **Intake**	Compliance with nutritional guidelines among specific sub-group of the population (pregnant women)	Global	**Objective measures:** 24-h urinary sodium excretion; anthropometric measurements (weight and height)<br><br>**Declarative data:** structured interviews conducted by trained field workers to collect socio-economic and demographic data.	Pregnant women of this study may be borderline iodine deficient. Findings highlight the need for ongoing monitoring of iodine status among vulnerable pregnant women considering the recently introduced salt reduction policy in South Africa.
	(118)<br>Wald and Leshem, 2003	ISR<br><br>*n* = 80<br><br>A random sample of students participating in scheduled aerobic or basketball sessions; 38 women (aged 23.12±0.29) and 42 men (aged 23.46±0.50)	To evaluate the effect of salt consumption on the flavour preference or aversion for a novel drink after exercise<br><br>→ **Preference**	Food physico-chemical interactions with the physiology of salt compensation	CAS	**Collected data using experiments:** four groups of 20 students practising sports performed 4 physical exercise sessions (aerobics and basketball) for 90 minutes at 2 - 3-day intervals. During the exercise, the participants were asked to drink 100 ml of root beer,	The exercises given salt supplements paired with the root beer preferred more flavour when they sweated much in comparison to those who sweated little.<br><br>The exercisers who sweated a lot experienced a conditioned flavour aversion to a novel

						and each of the groups received capsules containing salt. The amount of sweat was assessed by weighing and controlled by preserving the same temperature and hygrometry at the gym; the amount of water consumed during the sport session was taken into account. The preference for the unknown drink flavour was assessed on the basis of a 10 cm line rating with the word (disgusting on the left and excellent on the right). Familiarity of the taste, as well as the intensity of the flavour, were also measured.	drink when given the placebo pills (in comparison with controls). Those who sweated less during the sports sessions did not develop a taste preference for the drink during the course of the study, even when the pills ingested contained up to 600 mg NaCl.<br><br>The study suggests that the recovery of sodium lost during physical exercise can induce a rewarding physiological state, post-ingestive effects that condition food preferences.
